# Child Odors and Parenting: A Survey Examination of the Role of Odor in Child-Rearing

**DOI:** 10.1371/journal.pone.0154392

**Published:** 2016-05-03

**Authors:** Masako Okamoto, Mika Shirasu, Rei Fujita, Yukei Hirasawa, Kazushige Touhara

**Affiliations:** 1 Department of Applied Biological Chemistry, Graduate School of Agricultural and Life Sciences, The University of Tokyo, Tokyo, 113–8657, Japan; 2 ERATO Touhara Chemosensory Signal Project, JST, The University of Tokyo, Tokyo, 113–8657, Japan; 3 Technical Research Institute, Research & Development Center, T. Hasegawa Co., Ltd, Kanagawa, 211–0022, Japan; Instituto Cajal-CSIC, SPAIN

## Abstract

Parental caregiving is critical for the survival of our young and continuation of our species. In humans, visual and auditory signals from offspring have been shown to be potent facilitators of parenting. However, whether odors emitted by our young also influence human parenting remains unclear. To explore this, we conducted a series of questionnaire surveys targeting parents with children under 6 years old. First, we collected episodes on experiencing odors/sniffing various parts of a child’s body (*n* = 507). The prevalence of experiencing events described in those episodes was examined in a separate survey (*n* = 384). Based on those results, the Child Odor in Parenting scale (COPs) was developed, and subsequently used in the main survey (*n* = 888). We found COPs to have adequate content validity, concurrent validity, and reliability. Responses to the COPs demonstrated that parents, especially mothers with infants, are aware of odors from their offspring, and actively seek them in daily child-rearing. The factor structure and content of the COPs items indicated that child odors have both affective and instrumental roles. Affective experiences induce loving feeling and affectionate sniffing, while instrumental experiences pertain to specific hygienic needs. The head was the most frequent source of affective experiences, and the child’s bottom of instrumental. Each was experienced by more than 90% of the mothers with a child below 1 year of age. Affective experiences significantly declined as the child grew older, possibly associated with the decline of physical proximity between parents and child. This age-related decline was not prominent for instrumental experiences, except for the bottom, which significantly declined after 3 years of age. The present findings suggest that child odors play roles in human parenting, and that their nature and significance change during the course of a child’s development.

## Introduction

Parental caregiving is critical for the survival of our young, and the continuation of our species. Importantly, infants and children do not play a passive role in their care; rather, they are potent elicitors of caregiving behaviors. For example, infants and young children have facial features that tend to elicit perceptions of “cuteness” and the performance of caring behaviors from adults [1, 2]. Infants’ cries often prompt parents to pick them up [3]. While the influences of visual and auditory signals from the young have been the main focus in human parenting, in non-human species, odors emitted by the young have also been shown to play important roles in a range of parental behaviors, such as recognizing, accepting, and caring for offspring [4].

Interestingly, humans also share a capacity to utilize odors of their young when caring for them. Previous studies have repeatedly found that mothers were able to discriminate odors of their newborns from those of unfamiliar neonates (reviewed in [5]). Mothers tend to rate odors of neonates more favorably than non-mothers do [6], with reward systems of their brains being activated by the odor [7, 8]. Regarding later developmental stages, studies on parents with pre-pubescent and pubescent children also found that mothers [9, 10] and mothers and fathers [11] could recognize odors of their own child. Another study on pre-pubescent children found that parents who can recognize the odor of their own child tend to have more affectionate relationships with their children [12]. Those findings suggest the possibility that child odors play some role in human parenting, however, previous studies have predominantly focused on either neonates [5–8] or pre-school to school-age children [9–12]. Additionally, most of the previous studies that have examined this association have used clothing worn by offspring to evaluate odor samples [5–12]. Considering that humans emit odors from a wide range of sources, including various secretory glands and excretions [13], some of which likely change during the course of development, it is possible that effects of odors from different parts of the body, and from post-neonatal infants and toddlers were overlooked.

To more broadly explore the effects of child odor, the nature of the olfactory modality itself may be a big hurdle. For visual and auditory modalities, methodologies such as picture or auditory recordings to readily capture and reproduce stimuli are well established. In contrast, there are no such established methods for olfaction. Furthermore, methods suited to the collection of odor samples differ depending on the particular odor source. For example, while use of clothing may be appropriate for collecting odor from skin surfaces, collecting air samples during breathing, or bodily secretions, would likely be more suitable for other odor sources [14]. Considering these issues, it is presently not realistic to experimentally examine all of the possible odor sources at multiple developmental stages.

An alternative means of broad exploration might be to simply ask parents about their experiences with child odors using a questionnaire-based methodology. Questionnaire surveys are powerful tools for obtaining insights into the lives of people for a relatively low cost, and in a short period of time. If parents are commonly aware of odors from particular parts of their child’s body, odors from those body parts would be ecologically meaningful candidates for further examinations. Self-reports of various feelings and reactions to the odors would also help to infer the possible roles of child odors in parenting. In fact, questionnaires have been successfully used to broaden our views of the roles of odors in everyday living (reviewed in [15]), including body odors of families and partners [16–20].

Therefore, we used a questionnaire to explore how child odors might influence parenting. The development of a valid questionnaire is critical when using questionnaire survey methods. Following standard procedures [21], we first defined key concepts. Child odor was defined, based on literature on body odor [13, 22, 23], as odor emanating from a child’s body, bodily secretions, and excretions. Regarding the stages of child development, as mentioned earlier, studies on odors of human offspring have been limited to neonates (within several days after birth [6–8, 24–29]) and pre-school to school age children (majority of participants older than 6 years old [9–12] with an exception that included 3–5 years old as one of the target groups [10]). Animal studies have shown that odors from the young following the neonate stage [30, 31] continue to induce parental caregiving. Human studies focused on visual modality have indicated that not only infants but also children up to approximately 4.5 years of age have specific facial features that evoke feelings of protectiveness from adults [32]. Considering this, we chose parents with children under the age of 6 years as the target population.

**Fig 1:**
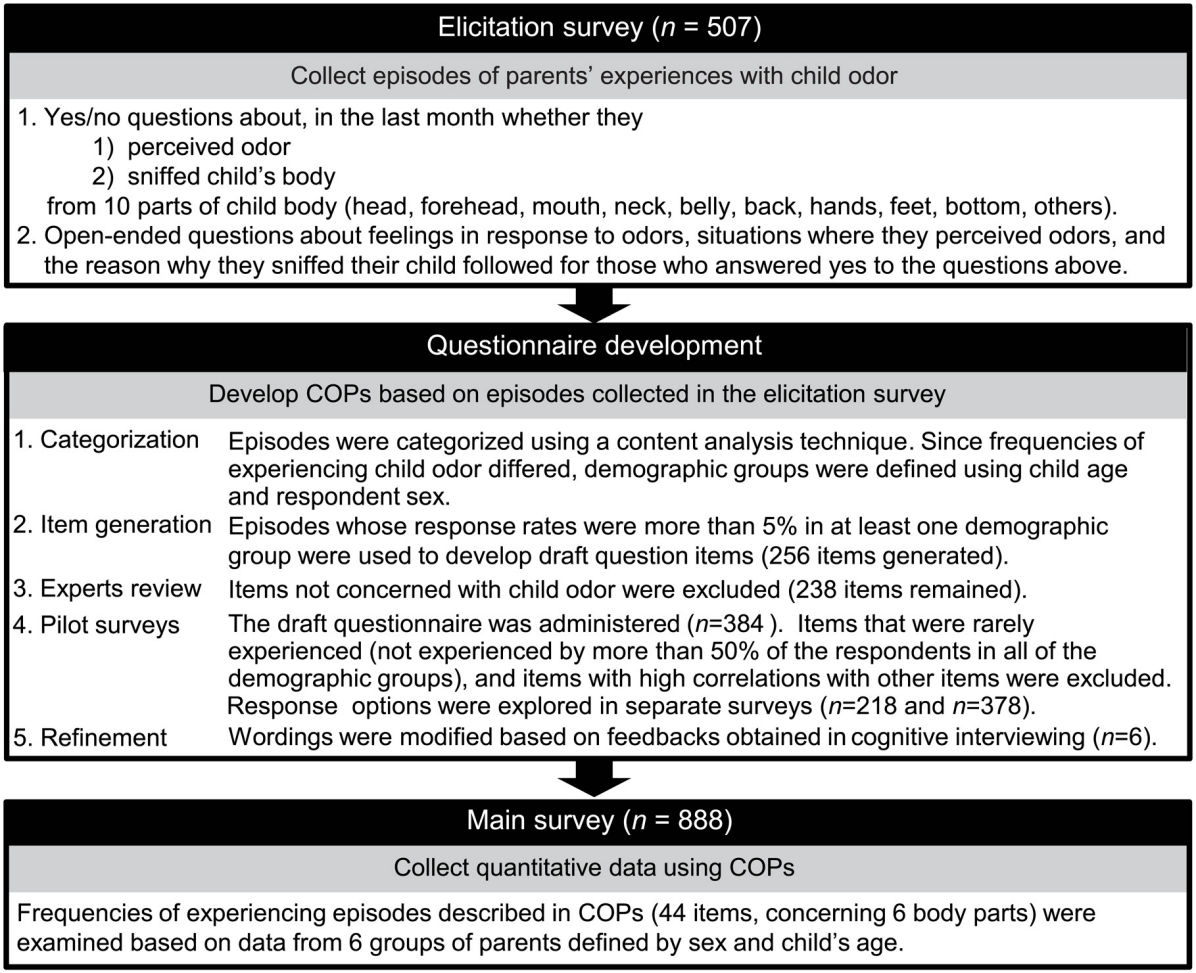
Steps undertaken to develop the Child Odor in Parenting scale (COPs). All the respondents were derived from the target population. None of the respondents participated in the survey more than once.

A questionnaire can be developed in either a theory-driven or exploratory manner [21]. As the topic of interest has yet to be studied in detail, we used the latter approach. Namely, we first collected episodes about experiences with child odors from our target population (Fig 1, upper box). Using those episodes, we developed the Child Odor in Parenting scale (COPs; Fig 1, middle box). After an examination of the reliability and validity of the COPs, data collected using the COPs (Fig 1, bottom box) were analyzed in order to answer the following questions: 1) Are parents aware of, and do they actively seek child odor in daily parenting? 2) What parts of a child's body are prominent odor sources? 3) Why do parents seek out odor, and what did they feel when perceiving an odor? 4) Do responses to questions 1 to 3 change over the stages of a child’s development? By answering these questions, we sought to explore whether child odors play a role in human parenting and, if so, their roles and sources.

## Results

### Elicitation and selection of question items for the COPs

In the elicitation survey (Fig 1, upper box, question 1), we found that frequencies of perceiving and actively seeking for child odor largely differed depending on the respondent’s sex, and on the age of the target child. Based on this observation, six demographic groups were defined; father/mother; child <1 year, 1–2 years, and 3–5 years. In order to avoid missing episodes that are important for a particular group, episodes that were mentioned by more than 5% of the respondents in at least one of the demographic groups were used to generate a draft of the questionnaire, which resulted in 256 initial items (Fig 1, middle box, step 2). After screening these items based on the content validity, understandability and the prevalence of experiencing the event described in each item (Fig 1 middle box, steps 3–5), 44 items across six body parts were retained for the COPs items (Fig 1 bottom box; Table 1). Items selected were similar across body parts, but there were also events specific to a body part, such as inferring what was ingested (mouth, MO05), sniffing while kissing (forehead, F05), and inferring excretion (bottom, B01, B03).

**Table 1:** Child Odor in Parenting scale (COPs).

*Instructions*		
We are studying how parents perceive and use odors from young children in daily child care. Please share your experiences by answering the following questions. Since this survey concerns odors arising from children themselves, please try not to include scents originating from cosmetics, such as shampoo.		
Did you experience the following events with your (youngest) child in the last month? Please mark most appropriate option: *No (0)*, *Less than once a week (1)*, *2–3 times a week (2)*, *Almost every day (3)*, *Everyday (4)*.		
Body parts	Item no.	Question items
Head	H01	Sniffed child's head because I like the smell
	H02	Sniffed child's head because he/she is cute
	H03	Sniffed child's head because it is soothing
	H04	Sniffed child's head because it smells good
	H05	Perceived odor from child's head and felt happy
	H06	Perceived odor from child's head and became soothed
	H07	Perceived odor from child's head and felt loving
	H08	Sniffed child's head without a particular reason
	H09	Perceived odor from child's head and thought it smelled good
	H10	Sniffed child's head naturally while interacting with child
	H11	Sniffed child's head because it was just in front of my nose
	H12	Sniffed child's head out of curiosity
	H13	Sniffed child's head to confirm it is not smelly
	H14	Sniffed child's head to check that it is clean
	H15	Perceived odor from child's head and thought "let me clean it"
Forehead	F01	Sniffed child's forehead naturally while interacting with child
	F02	Sniffed child's forehead because she/he is cute
	F03	Sniffed child's forehead without a particular reason
	F04	Sniffed child's forehead because it was just in front of my nose
	F05	Sniffed child's forehead while kissing
	F06	Perceived odor from child's forehead and became soothed
	F07	Perceived odor from child's forehead and felt loving
	F08	Perceived odor from child's forehead and thought it smells good
Hands	HA1	Perceived odor from child's hands and felt loving
	HA2	Perceived odor from child's hands and felt he/she is cute
	HA3	Sniffed child's hands because they are cute
	HA4	Sniffed child's hands naturally while interacting with child
	HA5	Perceived odor from child's hands and thought "let me clean them"
	HA6	Sniffed child's hands to confirm they were not smelly
Mouth	M01	Sniffed child's mouth naturally while interacting with child
	M02	Sniffed child's mouth because it was just in front of my nose
	M03	Sniffed child's mouth because she/he is cute
	M04	Perceived odor from child's mouth and felt loving
	M05	Perceived odor from child's mouth and knew what he/she ate
	M06	Perceived odor from child's mouth and thought "let me clean it"
	M07	Sniffed child's mouth to confirm it is not smelly
Neck	N01	Perceived odor from child's neck and felt loving
	N02	Sniffed child's neck naturally while interacting with child
	N03	Perceived odor from child's neck and thought "let me clean it"
Bottom	B01	Perceived odor from child's bottom and thought it is good that he/she had pooed/peed
	B02	Perceived odor from child's bottom and thought "let me clean it"
	B03	Sniffed child's bottom to see whether he/she pooed/peed
	B04	Perceived odor from child's bottom and found it smelly
	B05	Sniffed child's bottom to confirm it is not smelly

When administered, items regarding "sniff" and "perceive" were separated to avoid confusion. Order of the items was randomized within and between the body parts. As used here, "head" refers to scalp and hair, according to the typical usage of the original word in Japanese. Illustration indicating each body part was also attached for respondents for clarification.

### Factor structure

**Fig 2:**
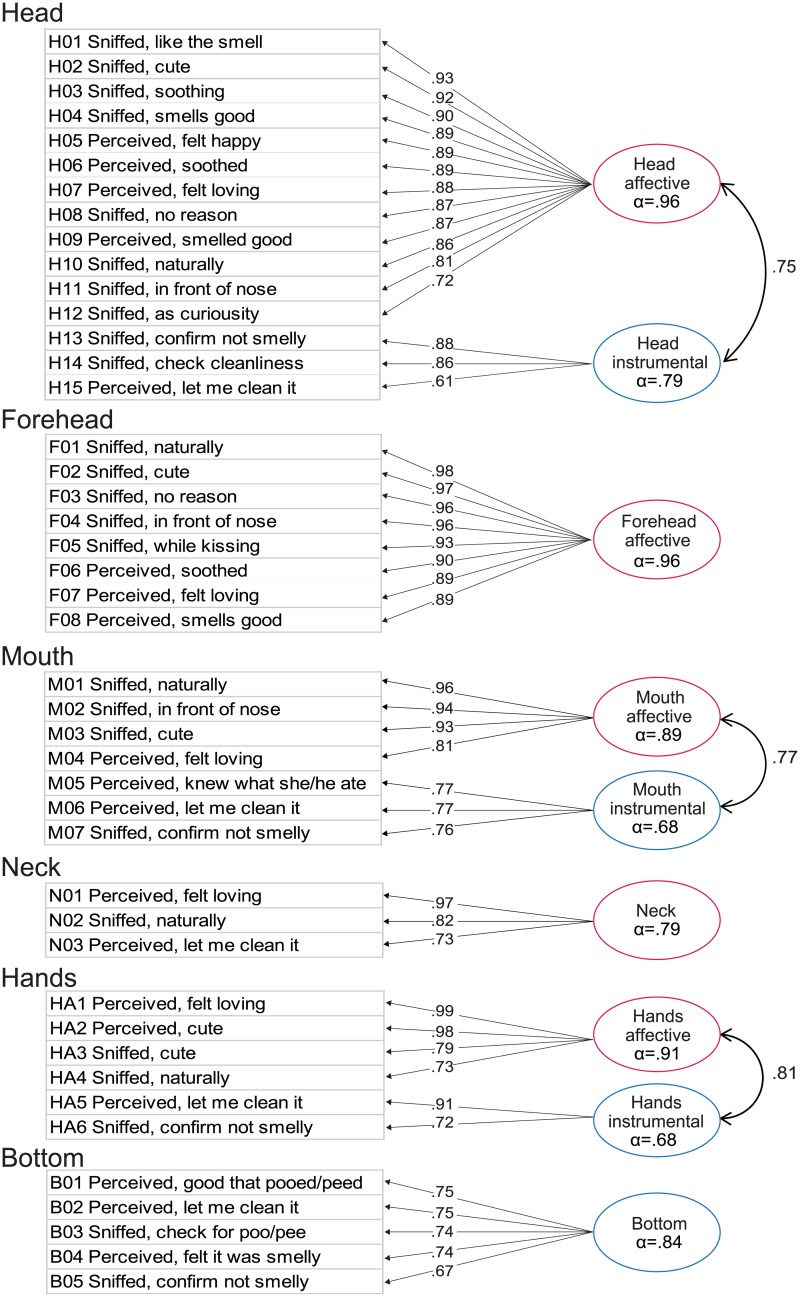
Factor structure of the Child Odor in Parenting scale (COPs) (*n* = 888). Abbreviated questionnaire items, standardized factor loadings, and correlation coefficients are presented for each body part. Item number corresponds to those shown in Table 1. Information on model fit for the selected models as well as for the alternative models is provided in S1 Table. Instrumental factors are shown in blue, and the Affective factors are in pink.

The factor structure of the COPs items was explored using data collected from the main survey (Fig 1, bottom box). In theory, odorants from different body parts can originate from different secretion/metabolic mechanisms, and have different chemical compositions [13, 23]. In addition, the frequency of experiencing episodes differed across body parts. Therefore, analysis was conducted for each body part separately. Since responses to some items had multimodal distributions, which could not be explained by demographic parameters, we used a Factor Mixture Modeling (FMM) framework [33]. As recommended [34], a series of alternative models were compared (S1 Table) to select the model with the best fit (Fig 2). Membership of items for each factor for each candidate model was determined using exploratory factor analysis conducted prior to FMM analysis [35].

For head, mouth, and hands, two factor models were selected, where items associated with affectionate responses such as “like the smell (H01)” loaded onto one factor, and practical responses such as “check cleanliness (H14)” loaded onto another factor (Fig 2). In the literature, caregiving behaviors are often described as affective or instrumental [3, 36]. Affective denotes affectionate care, such as stroking, patting, and palming the infants, while instrumental denotes physical care such as cleaning the baby, fixing its clothes, and changing diapers. Since the two factors we found for olfactory experiences seem to relate with those two categories, we labelled our factors “Affective” and “Instrumental”. From this perspective, the child’s Bottom contained only an Instrumental component, while the Forehead pertained only to an Affective component. The Neck had mixed components loading onto 1 factor, possibly because it did not contain a sufficient number of items to form two distinct and separate factors. Since the factor loading was highest for the affectionate item (N01) and lowest for the Instrumental item (N03), we interpreted the Neck subscale to primarily represent an Affective component. A high correlation between Affective and Instrumental factors found for the Head, Mouth, and Hands (Fig 2) indicated that the factors are not orthogonal. This is considered reasonable as both Affective and Instrumental components are important aspects of child care.

All the subscales defined by the factor structure demonstrated sufficient internal consistency as indicated by Cronbach’s alpha (Fig 2). Scores for each subscale were calculated by taking the mean of the items within each factor for each body part. Moderate positive correlations were found between scores for Instrumental subscales across different body parts, while strong positive correlations were observed for those of the Affective subscales (S2 Table). The present correlation pattern suggested that individuals who perceive and sniff odors from one body part tend to do so for other parts, especially for Affective reasons.

### Concurrent validity

Responses on the COPs may be susceptible to bias due to social desirability, as individuals may be reluctant to divulge body odors [37], or not to express positive feelings toward their child due to social expectations. If this is the case, the COPs scores would likely show an association with a measure of social desirability bias (MD-SDS [38, 39], see Table 2 for a list of measures). On the other hand, if the COPs is successful in assessing parents’ experiences with their child’s odor, scores are likely to correlate with factors directly influencing it, such as the respondent’s olfactory function and awareness (SAOQ [40], OAS [15], OELQ [20]) and level of involvement in child care (CCQ[41–44]). As shown in Table 3, none of the subscales exhibited a significant correlation with social desirability bias. Moreover, significant correlations were observed for measures of odor sensitivities and awareness, and level of involvement in child care. These correlation structures supported the discriminant and convergent validity of the COPs. Although we do not have a specific hypothesis about the relationship between demographic variables and the COPs scores, the absence of a significant correlation between the COPs scores and household income, and of significant correlations with child age and diet also indicated that the COPs measures constructs that are associated with odors arising from a child.

**Table 2:** Measures used to examine concurrent validity of the Child Odor in Parenting scale (COPs).

Measure	Description
Self-administered odor questionnaire (SAOQ)[40]	A self-report measure of olfactory acuity developed in Japan. Shown to be associated with odor recognition threshold determined by clinically used olfactory test. (α = .92, items = 20)
Odor Awareness Scale (OAS)*[15]	Measure of person's tendency to notice, pay attention to, or attach importance to odors in the environment. The Japanese version has three subscales. Reduced version of each subscale were used; awareness of positive odors (OAS-positive, α = .69, items = 3), awareness of negative odors (OAS-negative, α = .92, items = 4), and being affected by negative odors (OAS-nega-affected, α = .76, items = 3).
Odors in Everyday Life Questionnaire (OELQ)*[20]	Two of its subscales were used: Sexual role of bodily odor scale (OELQ-body; attracted, aroused, and soothed by body odors, α = .79, items = 4) and Ecological Odor Sensitivity scale (OELQ-ecological; sensitive to, aware of, and attentive to odors in daily life, α = .83, items = 5). Developer of OELQ found that, using sensory evaluation test, scores for those subscales to positively correlate with affective, and cognitive responsiveness respectively[20].
Child Care Questionnaire (CCQ)*	CCQ is prepared based on Parental Responsibility Scale [41, 42, 44]. Score for the original scale is shown to correlate with level of testosterone among fathers of young children[43]. In the CCQ, frequency of involving various child care activities, such as feeding child, playing with child, etc. were asked as a measure of level of involvements in child care (α = .94, items = 13).
Marlowe-Crowne Social desirability scale (MC-SDS) [38, 39]	Measure of social desirability bias, a tendency of respondents to answer questions in a manner that will be viewed favorably by others (α = .77, items = 22).

* Japanese versions were created for those that were not available in Japanese (S1 Text). α, Cronbach's alpha; items, number of items. Numbers in brackets indicate reference number.

**Table 3:** Correlation between scores for the Child Odor in Parenting scale (COPs) and related measures (*n* = 888).

	Head				Forehead		Mouth				Hands				Neck		Bottom	
Measure	Aff.		Inst.		Aff.		Aff.		Inst.		Aff.		Inst.		Aff.		Inst.	
Respondent characteristics																		
Olfaction-related scales																		
SAOQ	.25	***	.19	***	.27	***	.24	***	.25	***	.24	***	.20	***	.27	***	.19	***
OELQ_ecological	.35	***	.23	***	.28	***	.25	***	.21	***	.29	***	.23	***	.28	***	.21	***
OELQ_body	.34	***	.19	***	.31	***	.25	***	.20	***	.30	***	.22	***	.31	***	.20	***
OAS_positive	.41	***	.28	***	.32	***	.27	***	.27	***	.31	***	.25	***	.31	***	.26	***
OAS_negative	.25	***	.21	***	.26	***	.21	***	.19	***	.25	***	.23	***	.24	***	.19	***
OAS_nega_affected	.18	***	.09	*	.15	***	.11	***	.09	**	.12	***	.05		.15	***	.11	***
Parent-child relationship																		
CCQ	.33	***	.21	***	.23	***	.27	***	.24	***	.28	***	.27	***	.21	***	.33	***
Social desirability																		
MC-SDS	-.05		-.05		-.01		-.05		-.05		.02		-.06		.00		-.05	
Demographics																		
Respondent age	-.19	***	-.10	**	-.16	***	-.19	***	-.10	**	-.19	***	-.14	***	-.17	***	-.24	***
Respondent sex	-.26	***	-.18	***	-.17	***	-.23	***	-.21	***	-.21	***	-.22	***	-.13	***	-.19	***
Household income	-.06		-.05		-.01		-.03		-.01		-.05		-.02		-.06		-.06	
Child characteristics																		
Child age	-.22	***	-.04		-.24	***	-.17	***	.13	***	-.18	***	-.07	*	-.20	***	-.41	***
Child sex	.01		.06		-.01		.03		.07	*	.01		.03		.02		.05	
Child's current diet																		
Weaning status	-.17	***	.00		-.21	***	-.12	***	.15	***	-.14	***	-.05		-.16	***	-.29	***
Breast milk	.15	***	-.01		.18	***	.14	***	-.13	***	.16	***	.07		.17	***	.28	***

Spearman's correlation coefficients are shown. Dummy code for Sex, 0 = female, 1 = male. Weaning status is coded as 1 = pre-weaning (milk only), 2 = weaning (milk and solids), 3 = weaned (solids only). Breast milk, 0 = currently not taking breast milk, 1 = currently taking breast milk regardless of whether solid food is started or not.

*p < .05.

**p < .001.

***p < .0001.

See Table 2 for abbreviation and S3 and S4 Tables for descriptive statistics.

### Parents’ awareness and use of child odor in parenting

**Fig 3:**
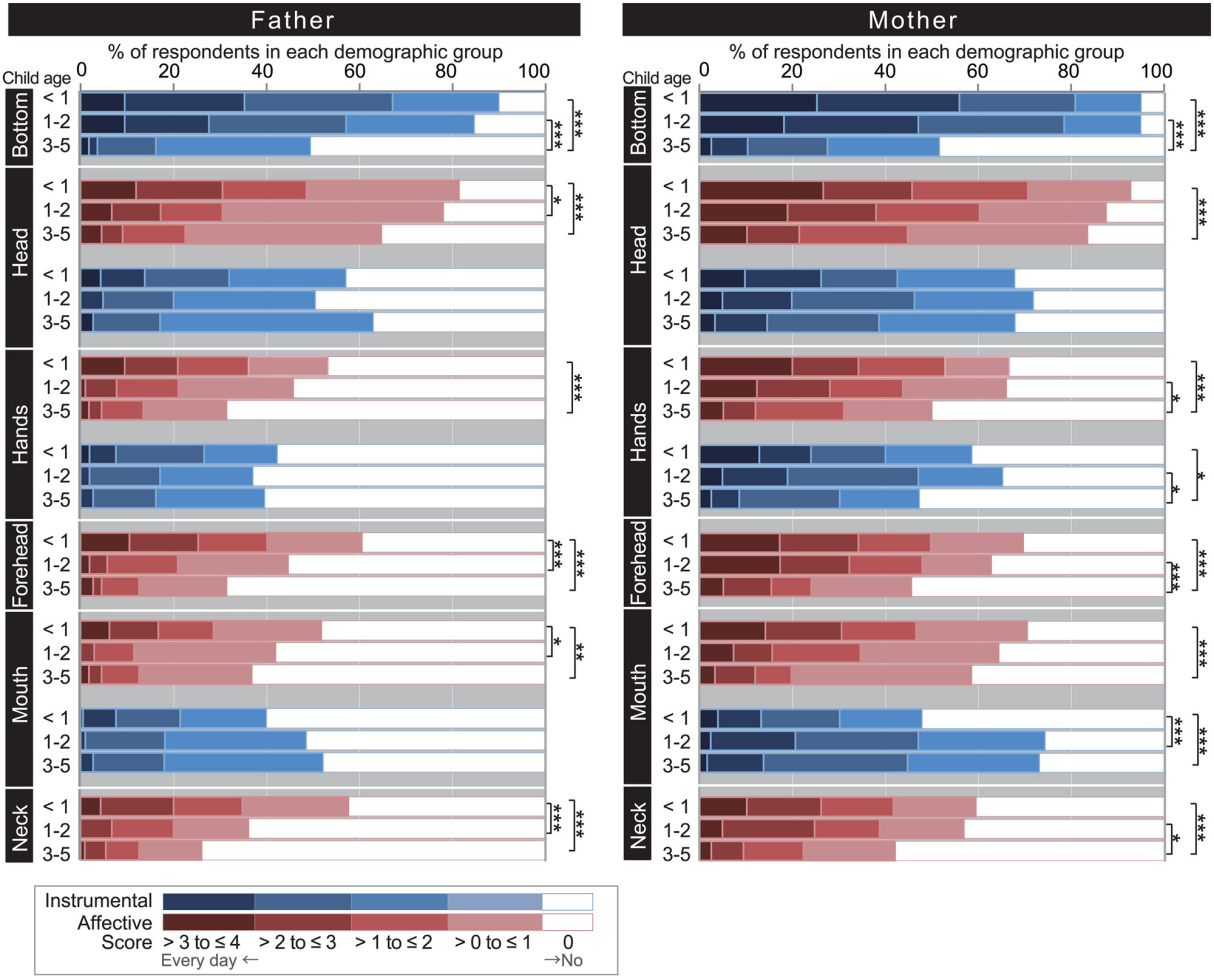
Cumulative bar chart showing differences in scores for the Child Odor in Parenting (COPs) subscales across child age groups. Scores on the COPs subscales are summarized for six demographic groups (i.e., parents’ sex and child age groups; total *n* = 888). Scores for subscales comprised of the Instrumental factor are shown in blue, and for the Affective factor in pink. Scores for subscales were calculated by averaging 5-point rating scores across items (0, no expreience; 4, everyday, see Table 1 for detail). The Kruskal–Wallis test followed by a pair-wise Mann-Whitney test with Bonferroni correction was used to examine differences among child-age groups within each sex (father/mother). *p < .05. **p < .01. ***p < .001. Comparisons between sex and body parts are shown in S1 and S2 Figs.

Fig 3 presents scores from the COPs subscales for each demographic group. Since scores from some of the subscales followed a multimodal distribution, we used cumulative bar charts to summarize the scores (see S4 Table for descriptive statistics). Frequencies of parent olfactory experiences differed across body parts, child age, and parent sex. Among groups with a child up to age 2, the bottom was the most frequent source of olfactory experiences (Fig 3 and S1 Fig). Specifically, parents use olfactory cues to check excretory status, with the frequency of this experience significantly lower among parents of a 3-5-year-old child. In Japan, the majority of children under age 3 have not completed toilet training [45]. They also lack sufficient linguistic skills to articulate their needs [46]. Given these conditions, odor is likely an important cue for parents to provide excretory care.

Affective experiences toward head odor were of similar frequency to those for the bottom (Fig 3 and S1 Fig). Among mothers of children up to age 2, about 90% had at least some experience in either having affectionate feeling towards head odor, or sniffing the head for affective reasons. For both fathers and mothers, Affective experience was highest among those with children under age 1 year. Among fathers, frequency becomes significantly lower above the age of 1 year, while the decline was more gradual among mothers. On the other hand, frequency of Instrumental use of head odor, e.g., sniffing the head to check for cleanliness, is generally lower than the Affective use, and there was no significant difference across child developmental stages.

Similarly, Affective experiences toward odors from the forehead, hand, and mouth are highest among parents of children under age 1 year, while Instrumental use generally did not exhibit significant differences across age (Fig 3). A prominent exception was the Instrumental use of mouth odor by mothers, which was significantly higher among those with a child above the age of 1, possibly coinciding with teething [47] and weaning [48]. The observed relationship between scores from the COPs subscales and child age provides another perspective on the categorization of parental odor experiences. Namely, a type of experience, which declines after the first few years of a child’s life (those described in the Bottom, Head-Affective, Hands-Affective, Forehead, Mouth-Affective, and Neck subscales), and those that remain over years (those described in the Head-Instrumental, Hand-Instrumental, and Mouth-Instrumental subscales). Categorizing the COPs subscales in this manner helps us in understanding differences in odor experiences among parents with a child in earlier (below 1–3 years old) and later (up to 6 years) stages of development.

Regarding sex differences, mothers generally have higher frequencies of olfactory behaviors compared to fathers, but the difference was not constant across child-age groups and types of experiences (S2 Fig). Among parents with children aged 2–3, mothers had significantly higher frequencies for all the olfactory experiences, while differences between mother and father was not that prominent among parents with a child below the age of 1 and above the age of 3. Together with the effect of child age, these results suggest that both fathers and mothers tend to have frequent child odor experiences when their child is young, with the decline of frequency being sooner and sharper among fathers than mothers.

### Regression analysis exploring predictors of the COPs scores

The differences found in COPs scores across demographic groups may be merely due to other factors associated with demographics (e.g., differences in the level of involvement in child care). In order to explore factors uniquely accounting for these COPs scores, we conducted stepwise multiple regression analysis. We focused only on two subscales that exhibited the highest experiences, Bottom and Head-Affective subscales, as error distributions of other subscales deviated from normal, which is undesirable for this type of analyses. Among the measures presented in Table 3, parameters that had meaningful correlations (ρ > 0.2)[49] with those particular subscales were used as initial predictors. There were some candidate predictors that were strongly correlated: respondent sex and the CCQ for the Head-Affective subscale, and child age, weaning status, and breast milk for the Bottom subscale (S5 Table). To avoid multicollinearity, we chose a variable that explained the greatest variances among those mutually correlated predictors for each subscale as the predictor; i.e., CCQ measure for Head-affective, and child age for Bottom. (Regarding the effect of breast milk, we conducted a separate exploratory examination, where we found positive correlations between Affective scores and continuous breast-feeding among mothers with infants in the weaning and post-weaning stages; see S2 Text and S6–S8 Tables).

Table 4 summarizes the results from the final model. For the Bottom subscale, child age was the strongest predictor followed by level of involvement in child care (CCQ) and individual tendencies in attending positive odor (OAS-positive). For the Head-Affective subscale, the OAS-positive, and the CCQ were the most important predictors, followed by the individual tendency in appreciating body odor (OELQ-body). Child age also uniquely accounted for the Head-Affective score.

**Table 4:** Final models predicting Bottom and Head-affective scores obtained by stepwise regression analysis (*n* = 888).

Independent variables	Bottom Inst.		Head Aff.	
Child age	-.42	***	-.16	***
OAS_positive	.17	***	.25	***
OELQ_body	-		.17	***
CCQ	.24	***	.25	***
Adjusted *R*^*2*^	.32	***	.27	***

Standardized beta coefficients are shown.

***p < .0001.

Abbreviations are the same as Table 2.

## Discussion

To the best of our knowledge, this is the first survey that examined parents’ experiences with odors of their children. To develop the questionnaire used in this study (the COPs; Table 1), we systematically collected relevant episodes from parents about experiences with odors from different parts of their child's body. We found COPs to have adequate content validity, concurrent validity, and reliability. From a sample of 888 parents with children under age of 6 years, responses to the COPs demonstrated that parents, especially mothers with infants, are aware of odors from their offspring, and actively seek them in daily child-rearing. The factors behind the responses examined using factor mixture modeling (Fig 2) indicated that parental experiences with child odors can be classified into two types: those associated with cleaning care (Instrumental factor; e.g., Perceived odor and thought “let me clean it”, Item B02), and those associated with affectionate care (Affective factor; e.g., perceived an odor and felt loving, Item H07). Frequent Affective experiences were characteristic of parents with children below ages of 1–3 years (Fig 3).

One of our aims was to explore the role of child odor in parenting. Experiences of child odor, expressed as COPs items (Table 1), and their factor structures (Fig 2) suggested two types of roles, Instrumental and Affective. Those two aspects fall in line with the role of infant cues studied along with Bowlby’s influential theory on parent-infant relationship [50, 51]. According to his theory, humans have an innate psycho-biological system called the “Caregiving System” that promotes caregivers to sensitively respond to infants’ needs to ensure their healthy development [50–52]. A body of empirical work on sensory signals from infants has also suggested two roles. The first conveying specific information about their needs, such as feeding, and the second evoking emotions from parents so as to increase their motivation for caregiving [3, 53, 54].

Thus far, studies examining these sensory cues have mostly focused on vision (e.g., smile), audition (e.g., cry), and touch [55–58]. In the case of olfaction, previous lab-based studies on infant odors have used neonates for odor sources and mainly focused on either recognition performance (reviewed in [5]) or brain activities evoked by the odors [7, 8]. Those studies indicated that the odor from neonates facilitated the recognition of offspring and may work as an enhancer of a mother’s positive affect and reactivity to the infant [36]. Our result was in line with those previous indications, and suggested that odors from older infants and children, not only neonates, have an influence on parents. Our results also suggest that olfaction plays each of the two roles important for parenting, i.e., to convey immediate needs, especially those related to cleaning, as typically seen in the Bottom-subscale of the COPs, and to evoke positive affection in parents as most often reported in the Head-Affective-subscale.

An interesting feature of olfactory cues appears to be their relation to proximity. The above-mentioned theory assumes another system named the “Attachment System”, which in conjunction with the Caregiving System, plays a prominent role in child development [50, 51, 59]. A key feature of the Attachment System is its strong power to motivate infants to seek proximity and physical contact to their primary caregivers, usually mothers. Even when infants have not developed sufficient motor skills to move towards caregivers, it is proposed that they can generate a variety of signals, such as crying, that makes caregivers more likely to approach them [53, 60]. According to the Affective experiences described in the COPs, one of the reasons for experiencing child odor was because the child’s body comes right up to their nose (e.g., Fig 2, Items H11, F04, M02). Parents also actively seek for odor due to its positive valence (e.g., Fig 2, Items H01, H03, H04). These episodes, along with Attachment theory, make us speculate about the possible role of child odors as a positive feedback agent: proximity makes caregivers perceive odor, and then odor inclines them to maintain proximity by motivating them to smell more.

From this perspective, the effect of a child’s age on parents’ Affective experiences is also suggestive. For all the body parts where Affective factors were found, frequencies of these experiences declined as the child grew older (Fig 3). For body parts where both Affective and Instrumental factors were found (i.e., head, hands, mouth), the effect of child age was not prominent for the Instrumental component. In other words, frequent Affective experiences were characteristic of parents with infants and toddlers below 3 years for mothers, and 1 year for fathers. Interestingly, the age-related decline could not be explained solely by a decline in the frequency of child care measured by the CCQ, as a child’s age uniquely explained variances in the Head-Affective score even after controlling for the CCQ (Table 4), and the CCQ demonstrated similar correlations with both the Affective and Instrumental subscales (Table 3).

With regard to the Attachment theory, studies on various cultures suggest that the level of proximity between a child and his/her primary caregiver, usually mothers, declines as the child grows older, with a large decrease in proximity occurring approximately around 3 or 4 years of age [53]. For example, lab-based studies in western culture found that while 1- and 2-year-olds tend to seek proximity with their mother in much the same way [54], older children increasingly depend on other distal strategies, such as eye contact and conversations, in place of physical contact [61]. An observational study of a hunter-gatherer society also found a decline in physical ties between infants and mothers at around 3 and 4 years of age [62]. It is interesting, and perhaps not surprising, that Affective experiences of parents on odor declines, as the proximity requirements of the child declines.

There are several possible explanations for this phenomenon. One such explanation is that odors that evoke Affective experiences require physical contact to be perceived, and therefore the Affective experience decreases when physical contact decreases. Another possible explanation is that the hormonal status of parents also changes according to their parental status (e.g., oxytocin exposure due to breast-feeding), which would influence their responsiveness to child cues [63]. Such changes in the physiological status of parents may influence their experiences with child odor. The positive correlations we found between Affective scores and continuous breast-feeding among mothers with infants in the weaning and post-weaning stage (S2 Text and S7 Table) provide some support for the two explanations presented above. Lastly, child odor itself will change over time, as body odors are influenced by diet, level of physical activity, activities of secretion glands, all of which changes within the age range of children we studied [13]. Regarding the type of odor, two possibilities should be considered: an individual’s signature odor that may convey kinship (reviewed in [5]), and odors common to all the young [6–8] that may act as olfactory “baby schema” [1]. Since the target population of the current study was biological parents, both interpretations are possible. Examination of these factors, together with child age and level of proximity is a topic for future studies.

Regarding the source of child odor, body parts that survived item screening of the COPs were all in the upper part of the body (Table 1), except for the bottom. This is probably because they are more likely approaching parents’ noses as described in items H11, F04, and M02 (Fig 2). Having those parts of an infants’ body close to the parents’ nose seems natural, considering humans’ biological nature in baby-rearing style: continuously carrying and feeding babies [60]. In addition, head, the most prominent source among them, has favorable conditions as an odor source [13]: the hair increases volatilization surface for odorous compounds; head is rich in sebaceous glands [64], which is an important source of body odor [22, 65]. Interestingly, anecdotes report that there is a distinctive “baby-head” scent [66]. While this has not been systematically studied, Russell et al. used the heads of neonates as odor sources and found that mothers could identify their own neonates using their odor [26]. Based on informal interviews with mothers, the authors suggested that breath might have been the discriminating odor cue [26]. Whether a child’s head produces a special scent, or is just acting as a reservoir of odors emanating from other parts of the body, is a subject for future study. Nevertheless, our results emphasize the necessity of examining odors from different body parts, other than the traditionally used odor source of infant-worn shirts.

While most of the parents with infants have at least some experiences with child odor, there were drastic individual differences: for example, among mothers with children younger than 1 year, nearly 20% reported having affective experiences with odors from children’s hands almost every day, but more than 30% reported no such experience (Fig 3, Hands-Affective). Such variability seems common with questionnaire studies on body odors. In a questionnaire survey of children’s olfactory experiences [18], 45% of the respondents reported that they were aware of natural body odors from relatives, even reporting feeling bothered upon losing them, while another 23% of respondents have never noticed the existence of such odors. In one questionnaire study of the odors of romantic partners, as many as 15% of women chose the maximum level of “often” regarding the frequency of intentionally smelling a partner’s clothing for affectionate reasons, while nearly the same percentage of women had never had such experiences. The percentage of non-experiencer was even higher among men (about 45%) in that study [17]. Although the score distribution was not reported in the original study of the OELQ-body [20], in the current survey, 23% of respondents chose “never experienced” to all the items on the OELQ-body. Such zero-inflation was not observed in other olfaction-related scales used here.

Causes of prominent “non-experiencer” in body odor questionnaires have not been clarified so far. Regarding the COPs, scores were correlated with those of other olfaction-related psychological scales, especially that of OAS-positive. The OAS-positive scores also accounted for the variance in COPs scores in regression analysis (Table 4). The OAS-positive is a psychological measure of a person’s tendency in attending to, and attaching importance to positive odors in their environment (Table 2). Scores for the OAS have also been associated with engagement in activities that requires olfaction (e.g., cooking), self-rated sense of smell, as well as performance on olfactory tests [15, 67]. Therefore, part of the variance in the COPs score is likely due to such olfaction-related psychobiological differences among respondents. As stronger odors are more likely to evoke attention [68], odors arising from a child are probably weak, at least for those who did not notice them. In addition, the OELQ-body uniquely explained the variance in the Head-Affective subscale score (Table 4). The OELQ-body is a psychological measure of a person’s tendency to be attracted to, aroused by, and soothed by natural body odor (Table 2). Therefore, psychological characteristics of individuals regarding the appreciation of body odor also accounts for different responses on the COPs. Considering the inter-individual variability in the biological basis of perception [69], and levels of secretions of the sources [70] of bodily odors, such biological factors may also be associated with individual differences in the responses to the COPs.

Finally, we found mothers to have higher frequencies than fathers in experiencing child odors. This is in line with literature in both the realm of olfaction, and in parental care: females tend to show better performance in olfaction tests [71, 72], and higher awareness of odors [15, 17, 18] than males, mothers tend to respond to child cues differently from fathers [73, 74]. However, in the current study, mothers were significantly more involved in child care than fathers (S3 Table), and it was difficult to separately evaluate the contributions of those factors. Statistically, association with COPs was stronger in the CCQ than for sex differences (Table 3). Regarding previous studies on odors of offspring, there are studies demonstrating that fathers, like mothers, are able to identify their own offspring by their odor [11, 12, 27] (although there are also conflicting reports [9, 26]). Regarding literature on parental care, emerging evidence suggests that fathers are able to develop responsiveness to infant, and its neural basis, through caregiving experiences [75–77]. Therefore, fathers may have the capability to respond to child odor similarly to mothers as long as they have similar levels of involvement in child care.

The current study has some limitations. First, this study is based on self-reports on subjective experiences. While subjective experiences are an important aspect in response to a stimulus, especially when emotion is involved [78], subconscious influence can at times be profound [79]. In a related vein, the child odors reported here were perceived under uncontrolled real-life conditions. Even though we asked respondents to try to exclude scents originating from cosmetics (Table 1), it might have been difficult to completely differentiate natural bodily odor from artificial odors. In addition, odors from parents could have attached to their children, and subsequently influenced their responses. Therefore, future studies using complementary approaches, such as behavioral and physiological measures taken under controlled conditions are needed to obtain a broader and a more accurate picture. Second, our study might have missed important, but infrequent experiences of child odor. In the current survey, we asked about parent’s experiences during the “past month” to reduce the influence of memory decay [80]. This made it difficult to capture experiences of rare events; for example, in the elicitation survey, there were episodes related to sickness (e.g., “perceived odor and thought he/she is sick”), but the number of participants who had this experience was not large enough for the item to survive screening. This may be because the probability of a child being sick in this specified time period was too small in frequency. Third, even though core components of parental care are expected to be universal in humans [81], cultural differences are known in its practices [3, 53]. Social structure also influences the degree of involvements in child care. Therefore, further cross-cultural studies are needed to test the universality of parental experiences with child odors.

In summary, the current study outlined parents’ daily experiences with odors from their offspring under the age of 6 years. We found that parents, especially mothers with infants were frequently aware of odors from their offspring, and actively sought them out for two predominant reasons: Affective, having affectionate feelings induced by perceiving their child’s odors, and Instrumental, checking for odors in order to clean their child. These observations, in light of relevant theories, suggested that child odors contribute to human parenting, by both increasing the positive feelings parents have with their children, and by conveying theirs needs of cleaning care. The fact that the Affective experiences were most frequent when the offspring is young also suggested a possible role of a child’s odor for maintenance of physical proximity between infants and parents. The results also identified the child’s bottom and head to be the odor sources that were most salient for parents, an important information for further studies involving collection and analysis of child odors. While studies of human olfaction are advancing, the functions of the olfactory system in day-to-day living, especially regarding odors from conspecifics, is largely understudied [82]. From the latter perspective, the current study adds an occasion where conspecific odors play a role in humans. Further investigations involving collection of child odors and direct examination of parental responses may clarify the effect of child odors on parental caregiving, as well as the role played by conspecific odors in humans.

## Method

### Development of the COPs

The overall procedure is summarized in Fig 1. To collect tangible episodes on various types of odors, questions were asked for 10 parts of a child’s body during the elicitation survey (Fig 1, upper box). When asking parents about their experiences with child odor, the time period was specified as “in the past month” to reduce the influence of memory decay [80] and changes due to child development. To minimize bias due to phrasing, we simply asked parents: whether they have perceived odor from their child, and whether they have actively sniffed their child. Open-ended questions followed for those who had experiences. Answers to the open-ended questions were coded in an exploratory manner using a content analysis technique [83] (Fig 1, middle box, step 1). First, a rater read through all of the responses and created categories. Next, two raters (M.O. and R. I.) independently categorized each response. New categories were created during this process. Third, the coding of the two raters was compared, and discrepancies were discussed. Finally, some categories with similar concepts were collapsed.

Draft questions were generated (Fig 1, middle box, step 2), and refined from three perspectives; content validity, psychometric property, and comprehensibility. Content validity was assessed by a panel of three experts with disciplines in chemistry, physiology, and psychology of olfaction (Fig 1; step 3 in the middle box). The experts reviewed all the draft questions and rated whether the items pertained to parents’ awareness and use of child odor according to our previously stated definition. The psychometric properties of items were assessed using data collected in the pilot survey (Fig 1, middle box, step 4). Frequency of experiencing events described in each item were rated using a five-point Likert scale (0 = no, to 4 = everyday). The comprehensibility of the phrasing was also evaluated. In this survey, we found that responses to most of the questions exhibited a bimodal distribution with one peak being at zero (not experienced). Bimodality was not explained by demographic properties, nor by changing response options (explored by separate surveys on selected items). Thus, we considered that having a bimodal distribution reflects the nature of parents’ experiences with child odor, and continued the assessment with nonparametric methods.

Items that are rated as difficult to understand by more than 10% of respondents in more than one demographic group, and those that are not experienced by more than 50% of the respondents in all of the demographic groups were excluded. Items with high associations (Spearman’s correlation coefficient, more than 0.8) with other items were also excluded to reduce redundancy. Understandability of the selected items (58 items) was ensured through cognitive interviewing [80], where respondents were asked to paraphrase questions (Fig 1; step 5 in the middle box). Finally, the main survey was conducted (Fig 1; bottom box). Using the same criteria regarding the frequency of experiences, 44 items were selected as the final COPs items.

### Respondents

Respondents for the elicitation survey, pilot surveys, and for the main survey were recruited from an on-line panel (Cross Marketing, Tokyo, Japan) using a quota sampling method. We used an internet survey methodology as the use of internet is common among our target population (more than 90% of the Japanese adult population ages 20–59 years had internet access at the time of the current study [84]). There were approximately 1.8 million people in the pool of participants. The target population was defined as those cohabiting with their biological child/children under the age of 6 years. Recruitment e-mails were randomly sent to whom registered information roughly matched the above criteria. For the main survey, we created 32 quotas defined by sex (father or mother), child’s sex (boy or girl), child age group (<6 months, 6–12 months, 1–2 years old, 3–5 years old) and parental experience (target child being the first-born or later-born). Respondents were recruited until the quota was filled. Since we did not find statistical differences in the scores for any of the COPs items between first-born and later-born children, between boys and girls, and among child age group of <6 months and 6–12 months, those groups were collapsed for all subsequent analysis. As the COPs specifically asks respondent’s experiences in the last month, those not seeing their children more than 15 days in the last month were excluded. We also set the following exclusion criteria in regard to olfaction: having self-reported problems with the olfactory system, pregnancy, smoking or cohabiting with smokers. Characteristics of the respondents from the main survey are summarized in S3 Table.

### Survey procedure

The survey was administered in two parts following standard procedure of the survey company: one part for demographic questions, and the other for the main survey questions. After consenting to participate, the respondents first answered the demographic questions. Next, for those meeting the demographic conditions, a brief introduction to the survey was given, and after a second consent protocol, they proceeded to the main questionnaire. In order to decrease a possible influence of volunteer bias [85], the fact that the survey was concerned with body odor was only revealed after the main survey was started. In the main survey, the COPs and other measures listed in Table 3 were included. In order to avoid any influence of other measures, the COPs, the main interest of the study, was asked first. The web pages used for on-line surveys were designed so that respondents could not proceed to the next question without completing the current one. Therefore, there were no non-response items. The elicitation, pilot, and main surveys were conducted in July 2013, April 2014, and October 2014, respectively.

### Factor mixture modeling

Factor mixture modeling (FMM) [33] was used to explore factor structures of the COPs subscales. FMM is a hybrid model integrating factor analysis (FA) and latent class modeling (LCA) [86]. FA serves to model unobservable theoretical concepts (e.g., “Affective caregiving”) based on observed variables (e.g., responses to COPs). Typically, FA assumes a homogeneous population; however, this assumption is sometimes violated. LCA deals with heterogeneity in a population by clustering participants into subpopulations. Thus, FMM, by combining LCA with CFA, enables fitting factor models to data from heterogeneous population. In the current analysis, we did not have any prior hypothesis about factor membership, number of factors, or classes. Therefore, we followed a model building strategy recommended for FMM that consists of five steps [34]. In the first step, FA and LCA were conducted separately. In the second step, FMM models assuming 1-factor and incremental number of classes were fit. Similarly, in the third step, FMM models assuming 2-classes and incremental numbers of factors were fit. The fourth step was the iterative process of increasing the number of classes and factors. Finally, in the fifth step, FA, LCA, and FMM models were compared to select the model with the best fit for the given data. Membership of items for each factor within each candidate model was determined using exploratory FA [35].

Analysis was conducted using Mplus version 7.3[87], treating dependent variables as ordered categorical variables. An exploratory FA was used to determine membership of items for each factor of candidate models, and a confirmatory FA was used for model comparisons. For exploratory FA, a weighted least squares means and variance adjusted (WLSMV) estimation method was used. For confirmatory FA and LCA, a maximum likelihood estimator with robust standard errors (MLR) was used. For FMM, MLR using a numerical integration was used. The relationship between a set of observed dependent variables and a set of categorical latent variables were described by logistic regression equations. We opted for a variant of an FMM in which only the factor means differed across latent classes.

Model fit information is listed in S1 Table, together with the basis of model selection for each body part. The best fit model among alternative models was selected primarily based on the Bayesian Information Criterion (BIC) index, while considering the result of the Lo-Mendell-Rubin test, entropy, correlation between factors, and interpretability [34]. While FMM simultaneously fit latent class and factor models to the data, we focused on the result from the FA, as our aim was to explore the factor structure of the COPs, rather than classification of respondents.

### Other statistical analysis

Reliability of the scales was evaluated using Cronbach’s alpha[88]. Concurrent validity was evaluated through correlation analysis between COPs scores and related variables described in Table 2. For statistical comparisons of variables where a normal distribution was not assumed, non-parametric tests were utilized. All significance tests were two-sided, and significance level was set at *p* < .05. Stepwise multiple linear regression analysis was used to identify predictors of the COPs score. The stepping criteria employed for entry and removal were based on the significance level of the F-value and set at .05 for alpha-to-enter and .1 for alpha-to-remove. The model that explained the maximum variance while having predictors with standardized β of greater than .15 was selected as the final model. IBM SPSS Statistics version 21 was utilized for all statistical analysis other than for the FMM, FA, and LCA analyses, which were conducted using Mplus version 7.3[87].

## Ethical considerations

The ethics committee of the University of Tokyo approved the protocol of the present study and all the survey procedures were carried out in accordance with the guidelines. The collection of on-line data complied with requirements specified in Japanese Industrial Standards “Personal information protection management systems—requirements” (JIS Q 15001). Written (electronic) consent was obtained from all the participants.

## Supporting Information

S1 Fig Differences in scores for the subscales of Child Odor in Parenting scale (COPs) across body parts. (PNG)

S2 Fig Differences in scores for the subscales of Child Odor in Parenting scale (COPs) between respondents’ sex. (PNG)

S1 Table Results of model comparison (n = 888). (DOCX)

S2 Table Correlations among the subscales of Child Odor in Parenting scale (COPs) (*n* = 888). (DOCX)

S3 Table Sample characteristics. (DOCX)

S4 Table Descriptive statistics of scores for the Child Odor in Parenting scale (COPs). (DOCX)

S5 Table Correlation matrix of all variables (*n* = 888). (DOCX)

S6 Table Sample characteristics for subgroups according to weaning stage. (DOCX)

S7 Table Correlation between scores for the Child Odor in Parenting scale (COPs) and current feeding types. (DOCX)

S8 Table Regression models predicting Bottom and Head-affective scores for mothers with post-weaning infants. (DOCX)

S1 Text Translation of psychological scales. (DOCX)

S2 Text Relationship of feeding type and the COPs scores within subgroups for different weaning stages. (DOCX)
