# Supplementary figures and images for: Child Odors and Parenting: A Survey Examination of the Role of Odor in Child-Rearing
Source: PLoS One. 2016 May 3;11(5):e0154392. doi: 10.1371/journal.pone.0154392 (PMC4854394; doi:10.1371/journal.pone.0154392)

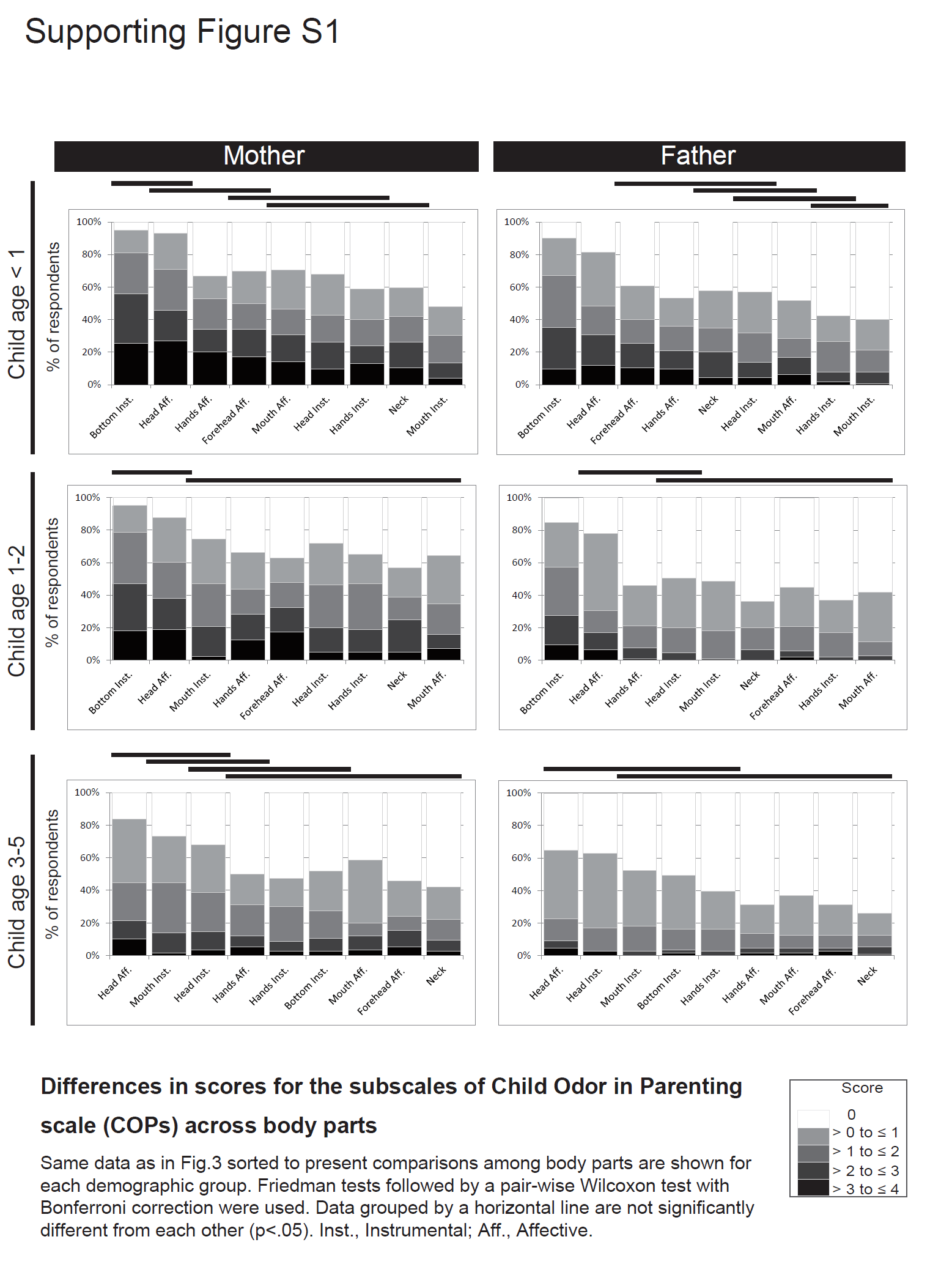

Supplement: S1 Fig — (PNG) [file pone.0154392.s001.png]

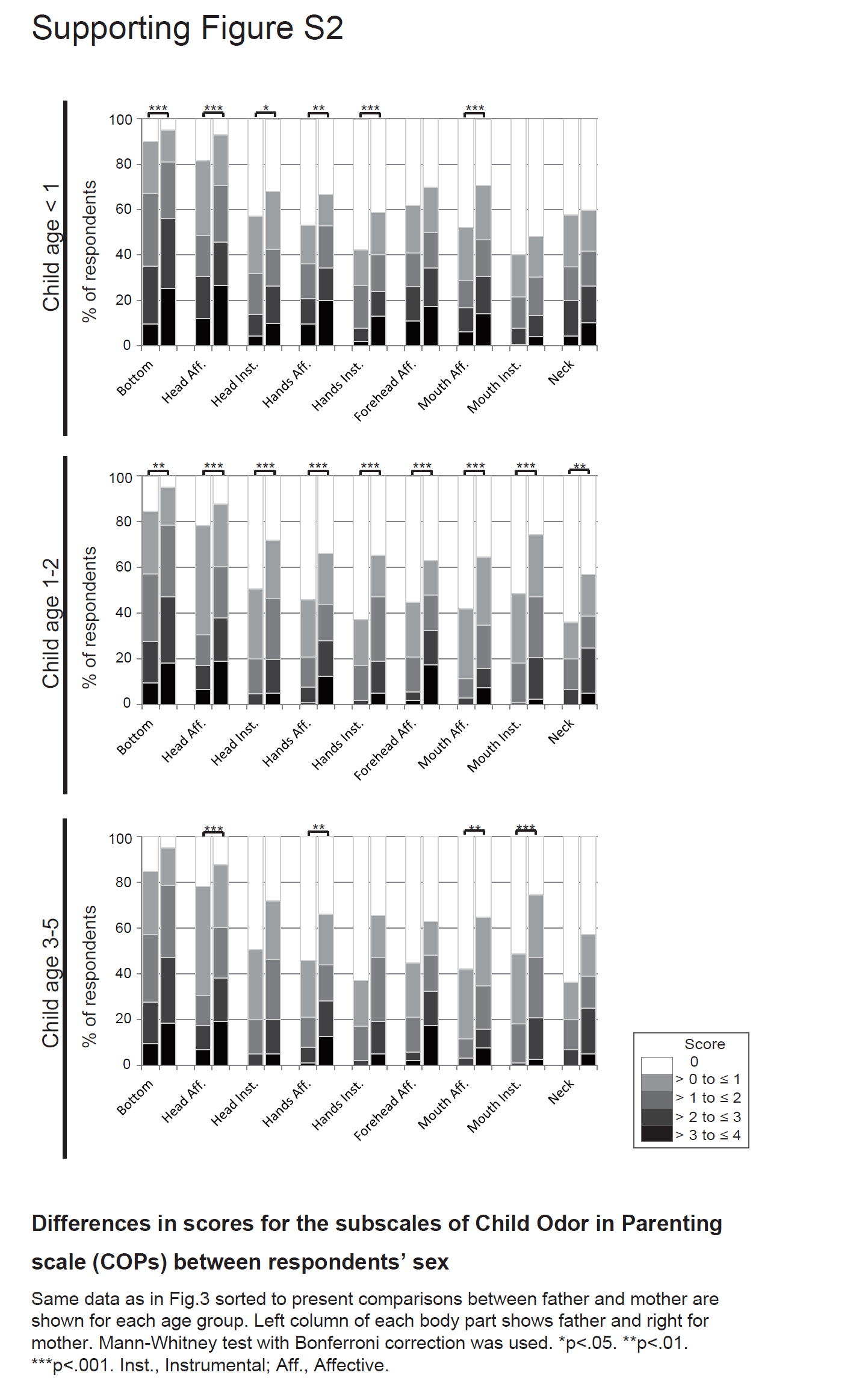

Supplement: S2 Fig — (PNG) [file pone.0154392.s002.png]
