# Supplementary material for: Child Odors and Parenting: A Survey Examination of the Role of Odor in Child-Rearing
Source: PLoS One. 2016 May 3;11(5):e0154392. doi: 10.1371/journal.pone.0154392 (PMC4854394; doi:10.1371/journal.pone.0154392)
Supplement: S1 Table — (DOCX) [file pone.0154392.s003.docx]

**S1 Table Results of model comparison (*n* = 888)**

|  | Model | | | Par. | LL | BIC | Entropy | LMR p-value |  | Comment |
| --- | --- | --- | --- | --- | --- | --- | --- | --- | --- | --- |
|  | Type | Class | Factor |  |  |  |  |  |  |  |
| *Head ( 15 items )* | | | |  |  |  |  |  |  |  |
|  | LCA | 2 | - | 121 | -15034 | 30889 | .97 | .00 |  |  |
|  | LCA | 3 | - | 182 | -13658 | 28551 | .96 | .00 |  |  |
|  | LCA | 4 | - | 243 | -12874 | 27397 | .96 | .77 |  | LMR suggested that a lower number of classes is sufficient |
|  | FA | 1 | 1 | 75 | -13372 | 27253 | - | - |  |  |
|  | FA | 1 | 2 | 76 | -13118 | 26753 | - | - |  |  |
|  | FA | 1 | 3 | 78 | -12733 | 25995 | - | - |  | Factors were highly correlated (.89) |
|  | FA | 1 | 4 | 81 | -12720 | 25990 | - | - |  | Factors were highly correlated (1.0) |
|  | FA | 1 | 5 | 85 | -12758 | 26093 | - | - |  | Factors were highly correlated (1.0); BIC increased beyond 5-factor |
|  | FMM | 2 | 1 | 77 | -13338 | 27198 | .98 | .00 |  |  |
|  | FMM | 3 | 1 | 79 | -13321 | 27178 | .92 | .01 |  |  |
|  | FMM | 4 | 1 | 81 | -13315 | 27181 | .81 | .04 |  |  |
|  | FMM | 5 | 1 | 83 | -13311 | 27185 | .63 | .09 |  | LMR suggested that a a lower number of classes is sufficient |
|  | FMM | 2 | 2 | 79 | -13079 | 26694 | .68 | .00 |  |  |
|  | **FMM** | **3** | **2** | **82** | **-13058** | **26672** | **.87** | **.02** |  | **Selected** |
|  | FMM | 4 | 2 | 85 | -13053 | 26684 | .58 | .01 |  |  |
|  | FMM | 2 | 3 | 82 | -12688 | 25932 | .72 | .00 |  | Factors were highly correlated (.89) |
|  | FMM | 3 | 3 | 86 | -12655 | 25895 | .76 | .02 |  | Factors were highly correlated (.93) |
|  | FMM | 4 | 3 | 90 | -12635 | 25881 | .85 | .01 |  | Factors were highly correlated (.93) |
|  | FMM | 2 | 4 | 86 | -12689 | 25962 | .67 | .00 |  | Factors were highly correlated (.99) |
|  | FMM | 3 | 4 | 91 | -12652 | 25922 | .80 | .08 |  | Factors were highly correlated (1.0) |
|  | FMM | 4 | 4 | 96 | -12602 | 25856 | .88 | .01 |  | Factors were highly correlated (1.0) |

**S1 Table (Cont.)**

|  | Model | | | Par. | LL | BIC | Entropy | LMR p-value |  | Comment |
| --- | --- | --- | --- | --- | --- | --- | --- | --- | --- | --- |
|  | Type | Class | Factor |  |  |  |  |  |  |  |
| *Forehead ( 8 items )* | | | |  |  |  |  |  |  |  |
|  | LCA | 2 | - | 65 | -6303 | 13048 | .96 | .00 |  |  |
|  | LCA | 3 | - | 98 | -5657 | 11979 | .96 | .00 |  |  |
|  | LCA | 4 | - | 131 | -5278 | 11446 | .96 | .77 |  | LMR suggested that a lower number of classes is sufficient |
|  | **FA** | **1** | **1** | **40** | **-5401** | **11074** | **-** | **-** |  | **Selected** |
|  | FA | 1 | 2 | 41 | -5033 | 10344 | - | - |  | Factors were highly correlated (.87)  Maximum number of factors extracted in exploratory factor analysis was 2 |
|  | FMM | 2 | 1 | 42 | -5387 | 11060 | .56 | .07 |  | Solution was not reliable (low entropy). |
|  | FMM | 2 | 2 | 44 | -5002 | 10303 | .48 | .00 |  | Solution was not reliable (low entropy). |
|  | FMM | 3 | 2 | 47 | -4987 | 10293 | .60 | .00 |  | Factors were highly correlated (.90) |
|  | FMM | 4 | 2 | 50 | -4975 | 10290 | .76 | .14 |  | LMR suggested that a lower number of classes is sufficient |
| *Neck ( 3 items )* | | |  |  |  |  |  |  |  |  |
|  | LCA | 2 | - | 25 | -2555 | 5279 | .87 | .00 |  |  |
|  | LCA | 3 | - | 38 | -2484 | 5226 | .86 | 1.00 |  | LMR suggested that a lower number of classes is sufficient |
|  | **FA** | **1** | **1** | **15** | **-2533** | **5168** | - | - |  | **Selected** |
|  | FMM | 2 | 1 | 17 | -2522 | 5160 | .58 | 1.00 |  | Solution was not reliable (class membership of class 2 became zero). |
| *Mouth ( 7 items )* | | | |  |  |  |  |  |  |  |
|  | LCA | 2 | - | 57 | -6054 | 12495 | .92 | .00 |  |  |
|  | LCA | 3 | - | 86 | -5693 | 11969 | .91 | .00 |  |  |
|  | LCA | 4 | - | 115 | -5538 | 11856 | .93 | 1.00 |  | LMR suggested that a lower number of classes is sufficient |
|  | FA | 1 | 1 | 35 | -5744 | 11725 | - | - |  |  |
|  | **FA** | **1** | **2** | **36** | **-5683** | **11611** | **-** | **-** |  | **Selected** |
|  | FA | 1 | 3 | 39 | -7164 | 14594 | - | - |  | BIC increased beyond 3-factor |
|  | FMM | 2 | 1 | 37 | -5725 | 11701 | .70 | .02 |  |  |
|  | FMM | 3 | 1 | 39 | -5698 | 11661 | .86 | .04 |  |  |
|  | FMM | 4 | 1 | 41 | -5694 | 11665 | .79 | .09 |  | LMR suggested that a lower number of classes is sufficient |

**S1 Table (Cont.)**

|  | Model | | | Par. | LL | BIC | Entropy | LMR p-value |  | Comment |
| --- | --- | --- | --- | --- | --- | --- | --- | --- | --- | --- |
|  | Type | Class | Factor |  |  |  |  |  |  |  |
| *Hands ( 6 items )* | | | |  |  |  |  |  |  |  |
|  | LCA | 2 | - | 49 | -5282 | 10897 | .93 | .00 |  |  |
|  | LCA | 3 | - | 74 | -4894 | 10291 | .95 | .00 |  |  |
|  | LCA | 4 | - | 99 | -4727 | 10126 | .95 | .67 |  | LMR suggested that a lower number of classes is sufficient |
|  | FA | 1 | 1 | 30 | -4892 | 9987 | - | - |  |  |
|  | FA | 1 | 2 | 31 | -4851 | 9912 | - | - |  |  |
|  | FA | 1 | 3 | 33 | -4850 | 9925 | - | - |  | BIC increased beyond 3-factor |
|  | FMM | 2 | 1 | 32 | -4871 | 9959 | .64 | .03 |  |  |
|  | FMM | 3 | 1 | 34 | -4827 | 9884 | .88 | .40 |  | LMR suggested that a lower number of classes is sufficient |
|  | **FMM** | **2** | **2** | **34** | **-4827** | **9885** | **.97** | **.01** |  | **Selected** |
|  | FMM | 3 | 2 | 37 | -4786 | 9823 | .87 | .23 |  | LMR suggested that a lower number of classes is sufficient |
| *Bottom ( 5 items )* | | | |  |  |  |  |  |  |  |
|  | LCA | 2 | - | 41 | -5701 | 11680 | .84 | .00 |  |  |
|  | LCA | 3 | - | 62 | -5394 | 11209 | .85 | .00 |  |  |
|  | LCA | 4 | - | 83 | -5227 | 11018 | .84 | .00 |  | 2-class-1-factor model was selected over this for interpretebility |
|  | LCA | 5 | - | 104 | -5153 | 11013 | .84 | 1.00 |  | LMR suggested that a lower number of classes is sufficient |
|  | FA | 1 | 1 | 25 | -5506 | 11181 | - | - |  |  |
|  | FA | 1 | 2 | 26 | -5506 | 11188 | - | - |  | BIC increased beyond 2-factor |
|  | **FMM** | **2** | **1** | **27** | **-5496** | **11175** | **.82** | **.00** |  | **Selected** |
|  | FMM | 3 | 1 | 29 | -5488 | 11174 | .79 | .06 |  | LMR suggested that a lower number of classes is sufficient |

LCA, latent class analysis; FA, factor analysis; FMM, factor mixture modeling; Class, number of classes; Factor, number of factors; Par., number of estimated parameters; LL, Log-likelihood; BIC, Bayesian Information Criterion; LMR, Lo-Mendell-Rubin test. Maximum number of classes and factors examined in FMM was defined based on the results of LCA and FA[[1](#_ENREF_1)]. Model selection should consider parameters listed above and interpretability of models[[1](#_ENREF_1), [2](#_ENREF_2)]. In the current analysis, the model with lowest BIC was selected for each body part among alternative models with reliable solutions. Models with low entropy (<.7), high correlation between factors (>.85), excessive number of classes according to LMR test (p >.05) were not selected. Regarding Bottom, 2-class-1-factor-FMM was selected over 4-class-LCA, because our primary aim was to explore factor structure of items, rather than classifying respondents.

**References**

1. Clark S, Muthén B, Kaprio J, D'Onofrio B, Viken R, Rose R. Models and strategies for factor mixture analysis: an example concerning the structure underlying psychological disorders. Structural Equation Modeling: A Multidisciplinary Journal. 2013;20(4):681703. doi: 10.1080/10705511.2013.824786.

2. Geiser C. Data Analysis with Mplus. 1 ed: The Guilford Press; 2012.
