# Supplementary material for: Child Odors and Parenting: A Survey Examination of the Role of Odor in Child-Rearing
Source: PLoS One. 2016 May 3;11(5):e0154392. doi: 10.1371/journal.pone.0154392 (PMC4854394; doi:10.1371/journal.pone.0154392)
Supplement: S2 Table — (DOCX) [file pone.0154392.s004.docx]

**S2 Table Correlations among the subscales of Child Odor in Parenting scale (COPs) (n = 888)**

|  |  |  |  |  |  |  |  |  |  |  |  |  |  |  |  |  |  |  |
| --- | --- | --- | --- | --- | --- | --- | --- | --- | --- | --- | --- | --- | --- | --- | --- | --- | --- | --- |
|  | Head | | | | Forehead | | Mouth | | | | Hands | | | | Neck | | Bottom | |
|  | Aff. | | Inst. | | Aff. | | Aff. | | Inst. | | Aff. | | Inst. | | Aff. | | Inst. | |
| Head Aff. | - | |  |  |  |  |  |  |  |  |  |  |  |  |  |  |  |  |
| Head Inst. | -^a^ | | - | |  |  |  |  |  |  |  |  |  |  |  |  |  |  |
| Forehead Aff. | .72 | ^***^ | .42 | ^***^ | - | |  |  |  |  |  |  |  |  |  |  |  |  |
| Mouth Aff. | .62 | ^***^ | .42 | ^***^ | .58 | ^***^ | - | |  |  |  |  |  |  |  |  |  |  |
| Mouth Inst. | .43 | ^***^ | .52 | ^***^ | .40 | ^***^ | -^a^ | | - | |  |  |  |  |  |  |  |  |
| Hands Aff. | .67 | ^***^ | .44 | ^***^ | .67 | ^***^ | .63 | ^***^ | .45 | ^***^ | - | |  |  |  |  |  |  |
| Hands Inst. | .48 | ^***^ | .56 | ^***^ | .45 | ^***^ | .47 | ^***^ | .50 | ^***^ | -^a^ | | - | |  |  |  |  |
| Neck | .59 | ^***^ | .45 | ^***^ | .66 | ^***^ | .56 | ^***^ | .45 | ^***^ | .67 | ^***^ | .50 | ^***^ | - | |  |  |
| Bottom Inst. | .49 | ^***^ | .42 | ^***^ | .48 | ^***^ | .46 | ^***^ | .32 | ^***^ | .48 | ^***^ | .43 | ^***^ | .46 | ^***^ | - | |

Spearman's correlation coefficients are shown. ***p<.0001. A correlation within each body part is estimated using factor mixture modeling (FMM) and shown in Fig.2.
