# Supplementary material for: Child Odors and Parenting: A Survey Examination of the Role of Odor in Child-Rearing
Source: PLoS One. 2016 May 3;11(5):e0154392. doi: 10.1371/journal.pone.0154392 (PMC4854394; doi:10.1371/journal.pone.0154392)
Supplement: S3 Table — (DOCX) [file pone.0154392.s005.docx]

**S3 Table Sample characteristics**

|  |  |  |  |  |  |  |  |  |  |  |  |  |  |  |  |  |  |
| --- | --- | --- | --- | --- | --- | --- | --- | --- | --- | --- | --- | --- | --- | --- | --- | --- | --- |
|  |  |  | **All** |  | Father | | | | | |  | Mother | | | | | |
|  |  |  |  |  | < 1 y.o. | | 1-2 y.o. | | 3-5 y.o. | |  | < 1 y.o. | | 1-2 y.o. | | 3-5 y.o. | |
|  |  | N | **888** |  | 210 | | 105 | | 111 | |  | 225 | | 121 | | 116 | |
| *Respondent characteristics* | | |  |  |  |  |  |  |  |  |  |  |  |  |  |  |  |
|  | Olfaction-related scales | |  |  |  |  |  |  |  |  |  |  |  |  |  |  |  |
|  |  | SAOQ (1-100) | **76±17.8** |  | 72.4±19.7 | ^a^ | 71.8±18.9 | ^a^ | 70.7±18.9 | ^a^ |  | 79.8±15.7 | ^b^ | 79.8±14.5 | ^b^ | 79.9±15.8 | ^b^ |
|  |  | OELQ_ecological (1-5) | **3.1±0.8** |  | 2.9±0.8 | ^a^ | 2.9±0.9 | ^a^ | 2.9±0.7 | ^a^ |  | 3.3±0.8 | ^b^ | 3.3±0.8 | ^b^ | 3.2±0.8 | ^ab^ |
|  |  | OELQ_body (1-5) | **2±0.9** |  | 2.1±1.0 | ^a^ | 2.0±0.9 | ^ab^ | 1.9±0.8 | ^ab^ |  | 2.0±1.0 | ^ab^ | 1.9±0.9 | ^ab^ | 1.7±0.8 | ^b^ |
|  |  | OAS_positive (1-5) | **3.1±0.9** |  | 3.0±0.9 | ^a^ | 2.9±0.9 | ^a^ | 3±0.7 | ^a^ |  | 3.3±0.8 | ^b^ | 3.3±0.8 | ^b^ | 3.2±0.9 | ^ab^ |
|  |  | OAS_negative (1-5) | **2.6±1** |  | 2.4±1.0 | ^a^ | 2.4±1.0 | ^ab^ | 2.5±1.0 | ^ab^ |  | 2.8±1.0 | ^ab^ | 2.7±1.1 | ^ab^ | 2.7±1.1 | ^b^ |
|  |  | OAS_nega_affected (1-5) | **3.9±0.8** |  | 3.8±0.8 | ^ab^ | 3.7±0.8 | ^a^ | 3.8±0.7 | ^ab^ |  | 4.0±0.7 | ^b^ | 4.0±0.7 | ^b^ | 4.0±0.8 | ^ab^ |
|  | Parent-child relationship | |  |  |  |  |  |  |  |  |  |  |  |  |  |  |  |
|  |  | CCQ (0-4) | **2.6±1** |  | 1.8±0.7 | ^ab^ | 1.9±0.7 | ^a^ | 1.6±0.7 | ^b^ |  | 3.5±0.3 | ^c^ | 3.5±0.4 | ^c^ | 3.1±0.5 |  |
|  | Social desirability | |  |  |  |  |  |  |  |  |  |  |  |  |  |  |  |
|  |  | MC-SDS (0-1) | **0.4±0.2** |  | 0.5±0.2 | ^a^ | 0.4±0.2 | ^a^ | 0.4±0.2 | ^a^ |  | 0.4±0.2 | ^a^ | 0.5±0.2 | ^a^ | 0.5±0.2 | ^a^ |
|  | Demographics | |  |  |  |  |  |  |  |  |  |  |  |  |  |  |  |
|  |  | Age (year) | **36.8±5.8** |  | 38.4±6 | ^b^ | 38.7±5.2 | ^b^ | 41±5.4 |  |  | 33.2±4.4 | ^a^ | 34.3±4.5 | ^a^ | 38.0±4.7 | ^b^ |
|  |  | Sex (% mother) | **52** |  | 0 | ^a^ | 0 | ^a^ | 0 | ^a^ |  | 100 | ^b^ | 100 | ^b^ | 100 | ^b^ |
|  |  | Household income (million yen) | **5.3±3.4** |  | 5.6±3.5 | ^a^ | 6.0±3.1 | ^a^ | 6.0±4.5 | ^a^ |  | 5.0±3.0 | ^b^ | 5.0±2.4 | ^b^ | 5.3±3 | ^ab^ |
| *Child characterisitics* | | |  |  |  |  |  |  |  |  |  |  |  |  |  |  |  |
|  |  | Age (month) | **21.3±19.3** |  | 5.7±2.7 | ^a^ | 22.2±6.7 | ^b^ | 50.9±8.8 | ^c^ |  | 5.7±2.8 | ^a^ | 22.3±6.6 | ^b^ | 50±8.7 | ^c^ |
|  |  | Sex (% girl) | **48.1** |  | 48.6 | ^a^ | 47.6 | ^a^ | 49.5 | ^a^ | # | 44.9 | ^a^ | 49.6 | ^a^ | 50.9 | ^a^ |
| *Child's current diet* | | |  |  |  |  |  |  |  |  |  |  |  |  |  |  |  |
|  |  | Pre-weaning (milk only; % yes) | **26** |  | 53 | ^a^ | 2 | ^b^ | 3 | ^b † †^ | | 48 | ^a^ | 2 | ^b^ | 2 | ^b † †^ |
|  |  | Weaning (milk & solids; % yes) | **40** |  | 47 | ^a^ | 53 | ^a^ | 24 | ^b^ |  | 52 | ^a^ | 42 | ^ab^ | 9 |  |
|  |  | Weaned (solids only; % yes) † | **33** |  | 0 | ^a^ | 44 | ^b^ | 72 | ^c^ |  | 0 | ^a^ | 55 | ^bc^ | 90 |  |
|  |  | Breast milk (% yes) | **58** |  | 85 | ^a^ | 48 | ^b^ | 23 | ^c^ |  | 89 | ^a^ | 38 | ^bc^ | 9 |  |
|  |  |  |  |  |  |  |  |  |  |  |  |  |  |  |  |  |  |

Abbreviations are the same as Table 2. Possible range of scores is shown in parenthesis for each scale. Score for the SAOQ was calculated according to the original developers. For other scales, scores were calculated as means of all the items. Scores and ages are shown as mean ± standard deviation (SD). Household income is shown as median ± interquartile range (IQR). One million yen was about 9,300 U.S. dollars at the time of the study. "Pre-weaning", those who have not started solid foods; "Weaning", taking both solid foods and breast milk or formula milk; "Weaned", those who are not taking breast milk or formula milk. "Breast milk", taking breast milk regardless of whether solid food is started or not. For each measure, differences between demographic groups were examined. For income, Mann-Whitney U test with Bonferroni correction was used. For frequency variables, Chi-squared test with Bonferroni correction was used. For others, one-way ANOVA with Tukey's HSD test was used. For each measure, summary statistics with same superscripts are not statistically different from each other (P<0.05). In general, females had higher scores in olfaction-related, and child care measures than males. For both genders, child care scores were lower as children are older. Respondent's age was higher in males than females, and those with elder child. † Here, "weaned" is defined as "not taking breast milk or formula milk". This definition is different from that used for weaning stage-wise analysis shown in S6-S8 Tables; see supporting text 2 for detail. ††Considering child age, "Pre-weaning" responses in 3-5 y.o. group are most likely due to respondents' misunderstanding.
