# Supplementary material for: Child Odors and Parenting: A Survey Examination of the Role of Odor in Child-Rearing
Source: PLoS One. 2016 May 3;11(5):e0154392. doi: 10.1371/journal.pone.0154392 (PMC4854394; doi:10.1371/journal.pone.0154392)
Supplement: S4 Table — (DOCX) [file pone.0154392.s006.docx]

**S4 Table Descriptive statistics of scores for the Child Odor in Parenting scale (COPs).**

|  |  |  | **All** | | | |  | Father | | | | | | | | | | | |  | Mother | | | | | | | | | | | |
| --- | --- | --- | --- | --- | --- | --- | --- | --- | --- | --- | --- | --- | --- | --- | --- | --- | --- | --- | --- | --- | --- | --- | --- | --- | --- | --- | --- | --- | --- | --- | --- | --- |
|  |  |  |  |  |  |  |  | <1 y.o. | | | | 1-2 y.o. | | | | 3-5y.o. | | | |  | <1 y.o. | | | | 1-2 y.o. | | | | 3-5y.o. | | | |
|  | ***n*** |  | **888** | | | |  | 210 | | | | 105 | | | | 111 | | | |  | 225 | | | | 121 | | | | 116 | | | |
| *HeadAff.* |  | Mean | **1.4** | **±** | **1.3** |  |  | 1.3 | ± | 1.3 |  | 0.9 | ± | 1.1 |  | 0.7 | ± | 0.9 |  |  | 2.0 | ± | 1.3 |  | 1.7 | ± | 1.3 |  | 1.3 | ± | 1.2 |  |
|  |  | Median | **1.1** | **±** | **2.2** |  |  | 1.0 | ± | 2.2 |  | 0.5 | ± | 1.2 |  | 0.3 | ± | 0.9 |  |  | 1.9 | ± | 2.3 |  | 1.6 | ± | 2.3 |  | 0.9 | ± | 1.7 |  |
| *HeadInst.* |  | Mean | **1.0** | **±** | **1.1** |  |  | 0.9 | ± | 1.1 |  | 0.6 | ± | 0.8 |  | 0.6 | ± | 0.8 |  |  | 1.2 | ± | 1.3 |  | 1.2 | ± | 1.1 |  | 1.0 | ± | 1.0 |  |
|  |  | Median | **0.6** | **±** | **1.5** |  |  | 0.4 | ± | 1.5 |  | 0.3 | ± | 1.0 |  | 0.5 | ± | 0.9 |  |  | 0.9 | ± | 2.1 |  | 1.0 | ± | 1.8 |  | 0.8 | ± | 1.5 |  |
| *ForeheadAff.* | | Mean | **1.0** | **±** | **1.3** |  |  | 1.1 | ± | 1.3 |  | 0.5 | ± | 0.8 |  | 0.4 | ± | 0.8 |  |  | 1.4 | ± | 1.4 |  | 1.3 | ± | 1.4 |  | 0.7 | ± | 1.1 |  |
|  |  | Median | **0.3** | **±** | **1.8** |  |  | 0.5 | ± | 2.0 |  | 0.1 | ± | 0.8 | ^†^ | 0.1 | ± | 0.3 | ^†^ |  | 1.0 | ± | 2.7 |  | 1.0 | ± | 2.4 |  | 0.1 | ± | 1.0 | ^†^ |
| *MouthAff.* |  | Mean | **0.9** | **±** | **1.2** |  |  | 0.8 | ± | 1.2 |  | 0.4 | ± | 0.6 |  | 0.4 | ± | 0.7 |  |  | 1.4 | ± | 1.4 |  | 1.0 | ± | 1.2 |  | 0.7 | ± | 0.9 |  |
|  |  | Median | **0.3** | **±** | **1.4** |  |  | 0.2 | ± | 1.4 |  | 0.2 | ± | 0.6 | ^†^ | 0.1 | ± | 0.4 | ^†^ |  | 1.0 | ± | 2.4 |  | 0.6 | ± | 1.6 |  | 0.3 | ± | 0.9 |  |
| *MouthInst.* |  | Mean | **0.8** | **±** | **0.9** |  |  | 0.5 | ± | 0.8 |  | 0.5 | ± | 0.6 |  | 0.5 | ± | 0.7 |  |  | 0.8 | ± | 1.1 |  | 1.2 | ± | 1.0 |  | 1.1 | ± | 0.9 |  |
|  |  | Median | **0.3** | **±** | **1.3** |  |  | 0.2 | ± | 1.0 | ^†^ | 0.3 | ± | 0.9 | ^†^ | 0.3 | ± | 0.9 |  |  | 0.3 | ± | 1.3 | ^†^ | 1.1 | ± | 1.7 |  | 1.0 | ± | 1.5 |  |
| *HandsAff.* |  | Mean | **1.0** | **±** | **1.3** |  |  | 1.0 | ± | 1.3 |  | 0.6 | ± | 0.9 |  | 0.4 | ± | 0.8 |  |  | 1.6 | ± | 1.5 |  | 1.3 | ± | 1.3 |  | 0.8 | ± | 1.1 |  |
|  |  | Median | **0.4** | **±** | **1.9** |  |  | 0.2 | ± | 2.0 |  | 0.2 | ± | 1.0 | ^†^ | 0.1 | ± | 0.4 | ^†^ |  | 1.4 | ± | 2.7 |  | 0.9 | ± | 2.2 |  | 0.2 | ± | 1.6 |  |
| *Hands Inst.* |  | Mean | **0.8** | **±** | **1.1** |  |  | 0.7 | ± | 1.0 |  | 0.5 | ± | 0.7 |  | 0.5 | ± | 0.7 |  |  | 1.2 | ± | 1.4 |  | 1.2 | ± | 1.1 |  | 0.8 | ± | 1.0 |  |
|  |  | Median | **0.4** | **±** | **1.6** | **^†^** |  | 0.3 | ± | 1.3 | ^†^ | 0.3 | ± | 0.8 | ^†^ | 0.3 | ± | 0.8 | ^†^ |  | 0.7 | ± | 2.1 |  | 1.1 | ± | 1.8 |  | 0.4 | ± | 1.6 | ^†^ |
| *Neck Aff.* |  | Mean | **0.9** | **±** | **1.1** |  |  | 1.0 | ± | 1.2 |  | 0.5 | ± | 0.8 |  | 0.3 | ± | 0.8 |  |  | 1.2 | ± | 1.3 |  | 1.0 | ± | 1.2 |  | 0.6 | ± | 1.0 |  |
|  |  | Median | **0.3** | **±** | **1.6** | **^†^** |  | 0.5 | ± | 1.8 |  | 0.2 | ± | 0.9 | ^†^ | 0.1 | ± | 0.3 | ^†^ |  | 0.7 | ± | 2.2 |  | 0.5 | ± | 2.0 |  | 0.2 | ± | 1.0 | ^†^ |
| *Bottom Inst.* |  | Mean | **1.5** | **±** | **1.2** |  |  | 1.6 | ± | 1.1 |  | 1.5 | ± | 1.1 |  | 0.5 | ± | 0.8 |  |  | 2.2 | ± | 1.1 |  | 2.0 | ± | 1.1 |  | 0.7 | ± | 0.9 |  |
|  |  | Median | **1.5** | **±** | **2.0** |  |  | 1.6 | ± | 1.6 |  | 1.3 | ± | 1.7 |  | 0.2 | ± | 0.8 | ^†^ |  | 2.3 | ± | 1.7 |  | 2.0 | ± | 1.6 |  | 0.2 | ± | 1.2 |  |

Mean ± standard deviation (SD) and Median ± interquartile range (IQR) are shown. † Zero was used instead of 25 percentile values for those where 25 percentile could not be defined given data.
