# Supplementary material for: Child Odors and Parenting: A Survey Examination of the Role of Odor in Child-Rearing
Source: PLoS One. 2016 May 3;11(5):e0154392. doi: 10.1371/journal.pone.0154392 (PMC4854394; doi:10.1371/journal.pone.0154392)
Supplement: S5 Table — (DOCX) [file pone.0154392.s007.docx]

**S5 Table Correlation matrix of all variables (*n* = 888)**

|  |  |  |  |  |  |  |  |  |  |  |  |  |  |  |  |  |  |  |  |  |  |  |  |  |  |  |  |  |  |
| --- | --- | --- | --- | --- | --- | --- | --- | --- | --- | --- | --- | --- | --- | --- | --- | --- | --- | --- | --- | --- | --- | --- | --- | --- | --- | --- | --- | --- | --- |
|  | SAOQ | | OELQ _eco | | OELQ _body | | OAS _posi | | OAS _nega | | OAS _negaA | | CCQ | | MC-SOS | | age | | sex | | income | | child  age | | child sex | | Weaning status | | Breast milk |
| *Olfaction-related scales* | |  |  |  |  |  |  |  |  |  |  |  |  |  |  |  |  |  |  |  |  |  |  |  |  |  |  |  |  |
| SAOQ | － |  |  |  |  |  |  |  |  |  |  |  |  |  |  |  |  |  |  |  |  |  |  |  |  |  |  |  |  |
| OELQ_ecological | .38 | ^***^ | － |  |  |  |  |  |  |  |  |  |  |  |  |  |  |  |  |  |  |  |  |  |  |  |  |  |  |
| OELQ_body | .15 | ^***^ | .28 | ^***^ | － |  |  |  |  |  |  |  |  |  |  |  |  |  |  |  |  |  |  |  |  |  |  |  |  |
| OAS_positive | .33 | ^***^ | .65 | ^***^ | .49 | ^***^ | － |  |  |  |  |  |  |  |  |  |  |  |  |  |  |  |  |  |  |  |  |  |  |
| OAS_negative | .36 | ^***^ | .68 | ^***^ | .20 | ^***^ | .40 | ^***^ | － |  |  |  |  |  |  |  |  |  |  |  |  |  |  |  |  |  |  |  |  |
| OAS_nega_affected | .20 | ^***^ | .38 | ^***^ | .09 | ^*^ | .39 | ^***^ | .27 | ^***^ | － |  |  |  |  |  |  |  |  |  |  |  |  |  |  |  |  |  |  |
| *Parent-child relationship* | |  |  |  |  |  |  |  |  |  |  |  |  |  |  |  |  |  |  |  |  |  |  |  |  |  |  |  |  |
| CCQ | .26 | ^***^ | .25 | ^***^ | .03 |  | .24 | ^***^ | .23 | ^***^ | .15 | ^***^ | － |  |  |  |  |  |  |  |  |  |  |  |  |  |  |  |  |
| *Social desirability* |  |  |  |  |  |  |  |  |  |  |  |  |  |  |  |  |  |  |  |  |  |  |  |  |  |  |  |  |  |
| MC-SDS | -.05 |  | -.06 |  | -.08 | ^*^ | -.07 |  | .04 |  | -.10 | ^*^ | .03 |  | － |  |  |  |  |  |  |  |  |  |  |  |  |  |  |
| *Respondent demographics* | |  |  |  |  |  |  |  |  |  |  |  |  |  |  |  |  |  |  |  |  |  |  |  |  |  |  |  |  |
| Respondent age | -.02 |  | -.08 | ^*^ | -.08 | ^*^ | -.11 | ^**^ | -.03 |  | -.08 | ^*^ | -.38 | ^***^ | .07 | ^*^ | － |  |  |  |  |  |  |  |  |  |  |  |  |
| Respondent sex | -.22 | ^***^ | -.20 | ^***^ | .06 |  | -.19 | ^***^ | -.14 | ^***^ | -.12 | ^***^ | -.81 | ^***^ | .01 |  | .39 | ^***^ | － |  |  |  |  |  |  |  |  |  |  |
| Household income | .05 |  | .03 |  | .04 |  | -.03 |  | .04 |  | .03 |  | -.17 | ^***^ | .04 |  | .25 | ^***^ | .21 | ^***^ | － |  |  |  |  |  |  |  |  |
| *Child demographics* |  |  |  |  |  |  |  |  |  |  |  |  |  |  |  |  |  |  |  |  |  |  |  |  |  |  |  |  |  |
| Child age | -.03 |  | -.03 |  | -.09 |  | -.08 | ^*^ | -.04 |  | .00 |  | -.09 | ^**^ | .01 |  | .27 | ^***^ | .01 |  | .04 |  | － |  |  |  |  |  |  |
| Child sex | .06 |  | .03 |  | -.02 |  | .04 |  | -.02 |  | .00 |  | .04 |  | .02 |  | .03 |  | -.01 |  | .05 |  | -.02 |  | － |  |  |  |  |
| *Child's current diet* |  |  |  |  |  |  |  |  |  |  |  |  |  |  |  |  |  |  |  |  |  |  |  |  |  |  |  |  |  |
| Weaning status | -.04 |  | -.05 |  | -.11 | ^*^ | -.08 | ^*^ | -.08 | ^*^ | .01 |  | -.03 |  | .02 |  | .17 | ^***^ | -.07 | ^*^ | .01 |  | .82 | ^***^ | .00 |  | － |  |  |
| Breast milk | .00 |  | .00 |  | .11 | ^*^ | .01 |  | .05 |  | -.04 |  | .06 |  | -.03 |  | -.16 | ^***^ | .05 |  | -.01 |  | -.62 | ^***^ | .02 |  | -.76 | ^***^ | － |
|  |  |  |  |  |  |  |  |  |  |  |  |  |  |  |  |  |  |  |  |  |  |  |  |  |  |  |  |  |  |

Spearman's correlation coefficients are shown. Dummy codes for Sex, 0=female, 1=male. Weaning status is coded as 1=pre-weaning (milk only), 2=weaning (milk and solids), 3=weaned (solids only). Breast milk, 0=currently not taking breast milk, 1=currently taking breast milk regardless of whether solid food is started or not. *p<.05. **p<.001. ***p<.0001. See Table 2 for abbreviation.
