# Supplementary material for: Child Odors and Parenting: A Survey Examination of the Role of Odor in Child-Rearing
Source: PLoS One. 2016 May 3;11(5):e0154392. doi: 10.1371/journal.pone.0154392 (PMC4854394; doi:10.1371/journal.pone.0154392)
Supplement: S6 Table — (DOCX) [file pone.0154392.s008.docx]

**S6 Table Sample characteristics for subgroups according to weaning stage**

|  |  |  |  |  |  |  |  |  |  |  |  |  |  |  |  |  |  |  |  |  |  |  |  |  |  |  |  |  |
| --- | --- | --- | --- | --- | --- | --- | --- | --- | --- | --- | --- | --- | --- | --- | --- | --- | --- | --- | --- | --- | --- | --- | --- | --- | --- | --- | --- | --- |
|  |  |  |  | Mother | | | | | | | | | | | |  | Father | | | | | | | | | | | |
|  |  |  |  | Pre-weaning | | | | Weaning | | | | Post-weaning | | | |  | Pre-weaning | | | | Weaning | | | | Post-weaning | | | |
|  |  | ***n*** |  | 108 | | | | 147 | | | | 190 | | | |  | 111 | | | | 131 | | | | 169 | | | |
| *Child characterisitics* | | |  |  |  |  |  |  |  |  |  |  |  |  |  |  |  |  |  |  |  |  |  |  |  |  |  |  |
|  |  | Age (month) |  | 3.6 | ± | 1.6 |  | 9.3 | ± | 3.8 |  | 40.7 | ± | 13.7 |  |  | 3.9 | ± | 1.7 |  | 9.7 | ± | 4.0 |  | 42.1 | ± | 13.9 |  |
|  |  | Sex (% girl) |  | 46 | | | | 44 | | | | 51 | | | |  | 50 | | | | 47 | | | | 49 | | | |
| *Child's current diet* | | |  |  |  |  |  |  |  |  |  |  |  |  |  |  |  |  |  |  |  |  |  |  |  |  |  |  |
|  |  | Formula milk (% yes) |  | 8 | | | | 14 | | | | 3 | | | |  | 7 | | | | 21 | | | | 4 | | | |
|  |  | Mixed (% yes) |  | 32 | | | | 23 | | | | 1 | | | |  | 50 | | | | 33 | | | | 15 | | | |
|  |  | Breast milk (% yes) |  | 59 | | | | 63 | | | | 13 | | | |  | 43 | | | | 46 | | | | 14 | | | |
|  |  | Solid food (% yes) |  | 0 | | | | 100 | | | | 98 ^†^ | | | |  | 0 | | | | 100 | | | | 98 ^†^ | | | |
|  |  |  |  |  |  |  |  |  |  |  |  |  |  |  |  |  |  |  |  |  |  |  |  |  |  |  |  |  |

"Pre-weaning", infants below age 10 months and not taking solid foods; "Weaning" infants below age 20 months and taking both milk and solid foods; "Post-weaning" all the children above 20 months. Note that the definition of weaning stage is different from that shown in S3 Table. See Supporting text 2 for details of grouping respondents. "Formula milk", those taking formula milk without breast milk; "Mixed", those taking both formula and breast milk; "Breast milk", those taking breast milk without formula milk; "Solid food", those taking solid food regardless of also taking formula or breast milk. †Considering child age, responses of not having solid foods in ≧20 month old group are most likely due to respondents' misunderstanding.
