# Supplementary material for: Child Odors and Parenting: A Survey Examination of the Role of Odor in Child-Rearing
Source: PLoS One. 2016 May 3;11(5):e0154392. doi: 10.1371/journal.pone.0154392 (PMC4854394; doi:10.1371/journal.pone.0154392)
Supplement: S7 Table — (DOCX) [file pone.0154392.s009.docx]

**S7 Table Correlation between scores for the Child Odor in Parenting scale (COPs) and current feeding types**

|  |  |  |  |  |  |  |  |  |  |  |  |  |  |  |  |  |  |  |  |  |
| --- | --- | --- | --- | --- | --- | --- | --- | --- | --- | --- | --- | --- | --- | --- | --- | --- | --- | --- | --- | --- |
|  |  |  | Head | | | | Forehead | | Mouth | | | | Hands | | | | Neck | | Bottom | |
| Measure | | | Aff. | | Inst. | | Aff. | | Aff. | | Inst. | | Aff. | | Inst. | |  |  | Inst. | |
| *Mothers* | | |  |  |  |  |  |  |  |  |  |  |  |  |  |  |  |  |  |  |
|  | *Pre-weaning* | |  |  |  |  |  |  |  |  |  |  |  |  |  |  |  |  |  |  |
|  |  | Type of milk | -.14 |  | .05 |  | -.09 |  | -.04 |  | -.03 |  | -.02 |  | .03 |  | .10 |  | .02 |  |
|  | *Weaning* | |  |  |  |  |  |  |  |  |  |  |  |  |  |  |  |  |  |  |
|  |  | Type of milk | .09 |  | .03 |  | .20 | * | .18 | * | .08 |  | .10 |  | .02 |  | .27 | *** | .04 |  |
|  | *Post-weaning* | |  |  |  |  |  |  |  |  |  |  |  |  |  |  |  |  |  |  |
|  |  | Currently taking breast milk | .26 | *** | .14 |  | .25 | *** | .15 | * | .14 |  | .18 | * | .16 | * | .17 | * | .25 | *** |
| *Fathers* | | |  |  |  |  |  |  |  |  |  |  |  |  |  |  |  |  |  |  |
|  | *Pre-weaning* | |  |  |  |  |  |  |  |  |  |  |  |  |  |  |  |  |  |  |
|  |  | Type of milk | -.12 |  | -.10 |  | -.23 | * | -.02 |  | -.09 |  | -.06 |  | -.11 |  | .03 |  | -.01 |  |
|  | *Weaning* | |  |  |  |  |  |  |  |  |  |  |  |  |  |  |  |  |  |  |
|  |  | Type of milk | -.03 |  | -.16 |  | -.07 |  | .04 |  | -.09 |  | -.06 |  | -.11 |  | -.01 |  | -.02 |  |
|  | *Post-weaning* | |  |  |  |  |  |  |  |  |  |  |  |  |  |  |  |  |  |  |
|  |  | Currently taking breast milk | -.03 |  | -.11 |  | .07 |  | -.05 |  | -.06 |  | .11 |  | .04 |  | .05 |  | .14 |  |

Spearman's correlation coefficients are shown. *p<.05. **p<.01. ***p<.001. "Pre-weaning", infants below age 10 months and not taking solid foods; "Weaning" infants below age 20 months and taking both milk and solid foods; "Post-weaning" all the children above 20 months. See Supporting text 2 for details of grouping respondents. Codings, "Type of milk", Formula=1, Mixed =2, Breast=3; "Currently taking breast milk", no=0, yes=1.
