# Supplementary material for: Child Odors and Parenting: A Survey Examination of the Role of Odor in Child-Rearing
Source: PLoS One. 2016 May 3;11(5):e0154392. doi: 10.1371/journal.pone.0154392 (PMC4854394; doi:10.1371/journal.pone.0154392)
Supplement: S8 Table — (DOCX) [file pone.0154392.s010.docx]

**S8 Table Regression models predicting Bottom and Head-affective scores for mothers with post-weaning infants**

|  |  |  |  |  |
| --- | --- | --- | --- | --- |
| Independent variables | Bottom  Inst. | | Head Affective | |
| Child age | -.54 | *** |  |  |
| OAS_positive | - |  | .32 | *** |
| OELQ_body | .19 | ** | .16 | * |
| Currently taking breast milk | - |  | .16 | * |
|  |  |  |  |  |
| Adjusted *R^2^* | .32 | *** | .22 | *** |

Multiple regression analysis was conducted to examine whether breastfeeding uniquely accounts for the COPs scores even after controlling for other factors. Analysis conditions and criteria used were the same as the stepwise multiple regression analysis presented in the main text. Only Head-affective and Bottom were analyzed because the error distributions of other subscales were not normal. Breastfeeding remained as a significant predictor for the Head-Affective subscale, but not for the Bottom subscale. Standardized beta coefficients are shown. *p<.05. **p<.01. ***p<.001. Coding, "Currently taking breast milk", no=0, yes=1. Abbreviations are the same as Table 2.
