# Supplementary material for: Child Odors and Parenting: A Survey Examination of the Role of Odor in Child-Rearing
Source: PLoS One. 2016 May 3;11(5):e0154392. doi: 10.1371/journal.pone.0154392 (PMC4854394; doi:10.1371/journal.pone.0154392)
Supplement: S1 Text — (DOCX) [file pone.0154392.s011.docx]

## **Supporting text 1**

## **Translation of psychological scales**

**Preparation of the Shortened Japanese Version of the Odor Awareness Scale (OAS)**

*Translation*

While a Japanese version of OAS was also developed by Nakano & Ayabe-kanamura [[1](#_ENREF_1)], it was unavailable when the current study was conducted. Therefore, we also translated the scale. Translation of the OAS [[2](#_ENREF_2)] measure items followed a forward-backward procedure [[3](#_ENREF_3)], in which independent professional translators were utilized for each translation process. Two raters (a Japanese-English bilingual and a native English speaker) evaluated the concordance between the back-translated items and the original items. Forward-backward translation was repeated until both raters assessed all the back-translated items as semantically concordant with the original. Subsequent to the translation process, several phrases from the items were revised for suitability within the Japanese context. Those changes were discussed with the original authors of the OAS in order to maintain the integrity of the original scale. Finally, the comprehensibility of the resultant phrasing was assessed by students and researchers recruited at the University of Tokyo (*n* = 11), and minor edits in phrasing were made.

*Statistical analysis*

To investigate the underlying factor structure of the measure, an exploratory factor analysis was conducted using principal components extraction with a promax rotation. The criteria for extracting factors were based on the following: (a) Kaiser’s rule[[4](#_ENREF_4)], (b) a scree test[[5](#_ENREF_5)], and (c) the interpretability of extracted factors[[6](#_ENREF_6), [7](#_ENREF_7)]. Internal consistency was evaluated based on Cronbach’s α. Concurrent validity was evaluated using Spearman’s correlation coefficient with other related measures.

*Creation and evaluation of shortened version of OAS*

Since we aimed to use the OAS in conjunction with many other measures, we created a shortened version of the OAS to lessen the burden on participants. First, responses to the full version of the OAS (22 items) were collected (*n* = 507) and analyzed. The original OAS has two subscales: one for positive odors (OAS-P) and one for negative odors (OAS-N), and each subscale exhibits a one-factor structure. As in the original OAS-P, one factor, which accounted for 42.6% of variance, was also identified for the Japanese version of OAS-P. For the Japanese version of the OAS-N, two factors, one presumably related to a tendency to notice odor, and the other related to a tendency to be affected by odor, were identified. These factors accounted for 34.8%, and 9.1% of the variance, respectively. To create a shortened version, items that had high factor loadings (factor loadings >.65) within each factor were selected. Hereafter, we refer to the shortened Japanese version of OAS-P as OAS-positive, that of the first factor of OAS-N (noticing of negative odors) as OAS-negative, and the second factor of OAS-N (affected by negative odor) as OAS-nega-affected.

The reliability and validity of the shortened Japanese version of the OAS were assessed using data collected from the main survey (*n* = 888). As expected, three factors were identified: the first was presumably related to the tendency for noticing negative odors (*n* items = 4, OAS-negative), the second was being influenced by negative odor (*n* items = 3, OAS-nega-affected) and the third was noticing positive odor (*n* items = 3, OAS-positive). These factors accounted for 43.4%, 17.4%, and 11.2% of the variance, respectively. All the subscales had sufficient reliability (see Table 2 in the main text). Concurrent validity was assessed by examining the correlation of the OAS measures with other olfactory measures and the social desirability index used in the main survey (*n* = 888). Since the OAS is a measure of olfactory awareness, we expected positive correlations with the measure of olfactory sensitivity (SAOQ), and Odors in Everyday Life Questionnaire (OELQ). Convergent validity was supported by positive correlations found between all the olfactory related measures (S5 Table; note that there were common question items between OAS and OELQ; one item each for OAS-positive and OAS-negative). In general, small to moderate correlations were observed between the OAS and other olfactory-related measures, suggesting that those are measuring overlapping, but distinguishable constructs. The discriminant validity was demonstrated by a low correlation coefficient between the OAS and the Japanese version of the Marlowe-Crowne Social desirability scale (MD-SDS[[8](#_ENREF_8), [9](#_ENREF_9)]; S5 Table).

**Preparation of the Japanese version of the Odors in Everyday Life Questionnaire (OELQ)[**[**10**](#_ENREF_10)**]**

*Translation*

Two subscales from the OELQ were translated: the Sexual role of bodily odor scale (OELQ-body) and the Ecological Odor Sensitivity scale (OELQ-ecological) (see Table 2 in the main text). Translation and statistical analysis procedures were similar to that of the OAS, except that the phrasing of the draft version of the questionnaire was checked by seven volunteers (students and researchers recruited at the University of Tokyo).

*Psychometric property*

The reliability and validity of the Japanese version of the OELQ was assessed using data collected from the main survey (*n* = 888). In line with the original version, two factors were identified: one was presumably related to sensitivity to ecological odors (*n* items = 4, OELQ-ecological), and the other was appreciation of bodily odors (*n* items = 4, OELQ-body, Table 2 of the main text). These factors accounted for 40.5%, and 22.0% of the variance, respectively. As described in the OAS section, concurrent validity was supported by positive correlations found between all the olfactory related measures and no significant correlation observed with the Social desirability measure (S5 Table).

**Preparation of the Child Care Questionnaire (CCQ)**

*Translation of the Parental Responsibility Scale[*[*11-14*](#_ENREF_11)*] (PRS)*

The CCQ is prepared based on the Parental Responsibility Scale[[11-14](#_ENREF_11)] (PRS). PRS is a 24-item questionnaire that asks the parent to designate, who has primary responsibility for 24 types of child care activities, such as feeding the child, disciplining the child, etc. The translation and statistical analysis procedures were similar to that described for the OAS. We omitted four items, where behaviors described in them were not commonly performed in Japan according to the pilot data (e.g., use of a baby sitter; more than 30% not experienced, *n* = 888).

*Preparation of the CCQ*

The CCQ was prepared by modifying the PRS from the following three perspectives: 1) the PRS is designed to measure balance of parental responsibility shared between mother and father. Since our interest was to measure frequency of conducting child care activities rather than balance between father and mother, response options were revised. The same response options (5-point) and target period (last month) as in the COPs (Table 1 in the main text) were used. Relatedly, two items not appropriate for the new response option (items related to vaccination and sickness) were removed; 2) the PRS includes items regarding parental decision making. Since we are interested in child care activities that involve physical interaction with the child, we omitted those seven items, which pertain to decisions about child care policy (e.g. “Determine appropriate clothes”, “Determine and implement discipline strategies”); and 3) We added two child care activities frequently mentioned in the pilot study (*n* = 507): Carry/hold/cuddle baby in arms (the single Japanese word “dakko” refers to carrying, holding, and cuddling), and Provide hygienic care of the child’s mouth.

*Psychometric property*

The reliability and validity of the Japanese version of the CCQ was assessed using data collected from the main survey (*n* = 888). A one-factor structure was suggested for the CCQ, where 60.4% of variance was explained by the first factor. Reliability was sufficient (Table 2 of the main text). Correlations between the CCQ and other measures were reasonable: a strong positive correlation (.89) was observed with the Japanese version of the PRS and the respondent’s sex, and no statistically significant correlation was observed with the social desirability measure (see S5 Table).

**References**

1. Nakano S, Ayabe-Kanamura S. The applicability of the odor awareness scale to ]apanese. Tsukuba Psychological Research. 2014;47:1-8.

2. Smeets M, A. M. , Schifferstein H, N. J. , Boelema S, R. , Lensvelt-Mulders G. The odor awareness scale: a new scale for measuring positive and negative odor awareness. Chem Senses. 2008;33(8):725-34. doi: 10.1093/chemse/bjn038.

3. Steiner DL, Norman GR. Health measurement scales. A practical guide to their development and use 3ed. New York: Oxford University Press; 2003.

4. Kaiser HF. The application of electronic computers to factor analysis Educational and Psychological Measurement. 1960;20(1):141-51.

5. Cattell RB. The scree test for the number of factors. Multivariate Behavioral Research. 1966;1(2):245-76.

6. Tabachnick BG, Fidell LS. Using multivariate statistics. 5 ed. Boston: Allyn and Bacon; 2007.

7. Mertler CA, Vannatta RA. Advanced and multivariate statistical methods: practical application and interpretation. 3 ed: Pyrczak Publishing; 2005.

8. Kawauchi K. A study on social desirabilty scale score: examination of property as the concept of personality-Trait. The bulletin of the Rissho University. 2006;8:1-8.

9. Crowne D, Marlowe D. A new scale of social desirability independent of psychopathology. J Consult Psychol. 1960.

10. Cupchik G, Phillips K, Truong H. Sensitivity to the cognitive and affective qualities of odours. Cognition & Emotion. 2005;19(1):121-31. doi: 10.1080/0269993044100011.

11. McBride BA, Mills G. A comparison of mother and father involvement with their preschool age children. Early Childhood Research Quarterly. 1993;8(4):457–77.

12. Montague DPF, Walker-Andrews AS. Mothers, fathers, and infants: the role of person familiarity and parental involvement in infants' perception of emotion expressions. Child Dev. 2002;73(5):1339-52. doi: 10.1111/1467-8624.00475.

13. Mascaro J, Hackett P, Rilling J. Testicular volume is inversely correlated with nurturing-related brain activity in human fathers. Proc Natl Acad Sci U S A. 2013;110(39):15746-51. doi: 10.1073/pnas.1305579110.

14. Goodman S, Lusby C, Thompson K, Newport D, Stowe Z. Maternal depression in association with fathers' involvement with their infants: spillover or compensation/buffering? Infant Ment Health J. 2014;35(5):495-508. doi: 10.1002/imhj.21469.
