# Supplementary material for: Child Odors and Parenting: A Survey Examination of the Role of Odor in Child-Rearing
Source: PLoS One. 2016 May 3;11(5):e0154392. doi: 10.1371/journal.pone.0154392 (PMC4854394; doi:10.1371/journal.pone.0154392)
Supplement: S2 Text — (DOCX) [file pone.0154392.s012.docx]

## **Supporting text 2**

## **Relationship of feeding type and the COPs scores within subgroups for different weaning stages**

*Aim*

The current study was designed to broadly explore parental awareness of child odors across wide developmental stages, and not to examine the effect of feeding types in detail. However, as the influence of breast feeding on child odor, and that of parent-infant relationships are important topics [[1](#_ENREF_1)], we conducted a preliminary examination.

*Definition of subgroups according to weaning stage*

In regard to the effect of feeding types on scores for the COPs, correlational analysis using data from all respondents (Table 3) was not informative, as a child’s current diet (whether taking breastmilk, and whether weaned or not) was strongly correlated with the child’s age (Results section in the main text, S5 Table). Therefore, in order to examine the relationship between the COPs scores and feeding types in detail, we sought to create subgroups that contain relatively homogeneous respondents except for feeding type. To this end, we categorized respondents according to weaning stage and parents’ sex (S6 Table).

Regarding the start of weaning, categorization was according to the subjects' responses on the child’s current diet. We added an age restriction using data from the Japanese national survey [[2](#_ENREF_2)] to exclude outliers. Regarding the end of weaning, we used the definition in the Japanese national survey as "obtaining most of the energy and major nutrients from solid food, regardless of whether breast/bottle feeding has ceased or not". Since we did not pose this question to our respondents, we used child age, where more than 98% of infants met these criteria in the Japanese national survey [[2](#_ENREF_2)]. Thus in the current analysis, the definition for the weaning stage used for grouping respondents was as follows: "Pre-weaning", infants below age 10 months and not consuming solid foods; "Weaning" infants below age 20 months and consuming both milk and solid foods; "Post-weaning" all children older than 20 months. Characteristics of the respondents for each subgroup are summarized in S6 Table. The percentage of breast feeding, and the age of weaning found in the current survey were very close to those reported in a national survey for the Japanese population [[2](#_ENREF_2)].

*Results and discussion*

By reclassifying respondents according to child weaning stages and parents’ sex, the strong correlation between feeding type and child age mostly disappeared (Spearman’s correlation coefficients ranged between -.2 to .08). S7 Table presents correlations between feeding types and COPs scores for each weaning stage. Results of stepwise multiple regression analysis for Head-affective and Bottom are shown in S8 Table.

Most of the significant relationships found were for mothers with weaning and post-weaning children, where breast feeding positively correlated with the scores for the Affective subscales (note that “post-weaning” for this analysis was defined as “obtaining most of the energy and major nutrients from solid food”; thus, approximately 20% of children in this group still consumed breast milk. See S6 Table). Lack of effect in the pre-weaning stage may indicate that odors of very young infants lead to olfactory experiences in parents regardless of feeding type. However, as more than 90 % of pre-weaning infants were consuming breast milk (as also shown in the Japanese national survey, [[2](#_ENREF_2)]; S6 Table), the interpretation requires some care. Different effects of breastfeeding on mothers and fathers may indicate that the observed effects were associated with the act of breast-feeding rather than possible differences in child odor due to feeding type.

**References**

1. Rilling JK. The neural and hormonal bases of human parental care. Neuropsychologia. 2013;51(4):731-47. doi: 10.1016/j.neuropsychologia.2012.12.017.

2. Japanese Ministry of Health, Labour and Welfare. National growth survey on preschool children. 2010. Available: http://www.mhlw.go.jp/toukei/list/73-22.html . Accessed 4 Nov 2015.
